# Transgender identity in young people and adults recorded in UK primary care electronic patient records: retrospective, dynamic, cohort study

**DOI:** 10.1136/bmjmed-2023-000499

**Published:** 2023-11-28

**Authors:** Douglas Gordon John McKechnie, Elizabeth O'Nions, Julia Bailey, Lorna Hobbs, Frank Gillespie, Irene Petersen

**Affiliations:** 1 Department of Primary Care and Population Health, University College London, London, UK; 2 Division of Psychology and Language Sciences, University College London, London, UK; 3 Gender Identity Development Service, Tavistock and Portman NHS Foundation Trust, London, UK; 4 Patient author, London, UK

**Keywords:** Epidemiology, Mental health, Gender

## Abstract

**Objectives:**

To quantify the change in proportion of young people and adults identified as transgender in UK primary care records and to explore whether rates differ by age and socioeconomic deprivation.

**Design:**

Retrospective, dynamic, cohort study.

**Setting:**

IQVIA Medical Research Data, a database of electronic primary care records capturing data from 649 primary care practices in the UK between 1 January 2000 and 31 December 2018.

**Participants:**

7 064 829 individuals aged 10-99 years, in all four UK countries.

**Main outcome measures:**

Diagnostic codes indicative of transgender identity were used. Sex assigned at birth was estimated by use of masculinising or feminising medication and procedural/diagnostic codes.

**Results:**

2462 (0.03%) individuals had a record code indicating a transgender identity. Direction of transition could be estimated for 1340 (54%) people, of which 923 were assigned male at birth, and 417 were assigned female at birth. Rates of recording in age groups diverged substantially after 2010. Rates of the first recording of codes were highest in ages 16-17 years (between 2010 and 2018: 24.51/100 000 person years (95% confidence interval 20.95 to 28.50)). Transgender codes were associated with deprivation: the rate of the first recording was 1.59 (95% confidence interval 1.31 to 1.92) in the most deprived group in comparison with the least deprived group. Additionally, the rate ratio of the proportion of people who identified as transgender was 2.45 (95% confidence interval 2.28 to 2.65) in the most deprived group compared with the least deprived group. Substantial increases were noted in newly recorded transgender codes over time in all age groups (1.45/100 000 person years in 2000 (95% confidence interval 0.96 to 2.10) to 7.81/100 000 person years in 2018 (6.57 to 9.22)). In 2018, the proportion of people with transgender identity codes was highest in the age groups 16-17 years (16.23 per 10 000 (95% confidence interval 12.60 to 20.57)) and 18-29 years (12.42 per 10 000 (11.06 to 13.90)).

**Conclusion:**

The rate of transgender identity recorded in primary care records has increased fivefold from 2000 to 2018 and is highest in the 16-17 and 18-29 age groups. Transgender diagnostic coding is associated with socioeconomic deprivation and further work should investigate this association. Primary and specialist care should be commissioned accordingly to provide for the gender specific and general health needs of transgender people.

**What is already known on this topic**

- Accurately determining the size and distribution of transgender populations is challenging, but important for service planning, specialist healthcare resource allocation, and clinician training
- The last study from the UK based on primary care records that attempted to estimate this size and distribution of transgender populations was in 1998
- Few studies have examined the association between socioeconomic deprivation and transgender identity at the population level

**What this study adds**

- Estimates of the rates and proportion of people with a record of transgender identity in UK primary care health records during 2000-18 were reported
- Rates of recorded transgender identity have increased in all age groups over time, and are highest in ages 16-29 years
- Recorded transgender identity was associated with living in areas of socioeconomic deprivation

**How this study might affect research, practice, or policy**

- Sufficient resources, support, and training must be provided to primary and specialist care to adequately meet the healthcare needs (general and gender specific) of transgender people
- Reasons for the association between socioeconomic deprivation (particularly parental deprivation) and rates of recorded transgender identity are unclear; further research should explore this association
- Recognition and recording of transgender identity in primary care records provides opportunities for observational research (eg, comorbid physical and mental health conditions, healthcare use, screening, and mortality)

## Introduction

Transgender, or trans, is an umbrella term that describes a diverse group of people whose gender identity differs from their sex assigned at birth.^1^ Some transgender people experience gender dysphoria—ie, distress stemming from the difference between their gender identity and sex assigned at birth—and some seek gender affirming medical care, such as hormone treatment and gender affirmation surgery.^2^ Some individuals may also seek out psychological support to help to explore their gender identity before (or in the process of) making decisions about physical interventions.

UK National Health Service (NHS) specialist gender identity clinics have, historically, not accepted self-referrals, and so individuals seeking gender affirming specialist care must first be referred by an NHS primary care clinician. Some clinics now do allow self-referral, but still require that patients are registered with an NHS primary care practice, with whom the service will share care.^3^ Primary care clinicians are expected by the UK’s medical regulator to refer such patients without delay and to work collaboratively with specialist gender services, including prescribing and monitoring gender affirming hormones on the basis of specialist recommendations, where appropriate.^4^ The number of referrals to specialist gender clinics has increased substantially in recent years, among children, adolescents,^5^ and adults.^6^ Waiting times for NHS gender clinics may stretch to several years.^7^ As a consequence, some people seek private gender care; although, the exact number that do so is unclear.^8 9^ Self-medication with hormone treatment, obtained from illicit or quasi-legal sources, may also be widespread among some groups of transgender people.^10^ Primary care clinicians may still, however, be involved in gender care for people accessing treatment outside of the NHS. Shared care requests from the private sector (for primary care to take over hormone prescribing) are common^4 11^; and the UK's medical regulator has suggested that clinicians might justifiably choose to take over bridging prescribing as a harm reduction measure for some people who are self-prescribing.^4^ Primary care also retains responsibility for providing general physical and mental healthcare for their transgender patients, and a need to take gender identity into account to ensure appropriate access to screening programmes, such as cervical screening for trans men.^12^

Many transgender people experience difficulties accessing appropriate medical care, whether that be gender care or general healthcare.^2 13^ Transgender people experience stigma, discrimination, exclusion, and harassment, including in healthcare settings.^2^ Transgender people may be reticent to disclose their gender identity to clinicians due to fear of stigmatisation: transgender people report encountering clinicians who deny the existence of transgender as a legitimate identity or refuse to refer them to specialist gender care.^14^

Estimates of the proportion of the population who identify as transgender vary, depending on the definition and method used. Studies based on surveys of general populations report much higher rates of self-identification as transgender, non-binary, gender diverse or gender questioning than those examining diagnostic coding of transgender identity in healthcare records.^15^ Among people who are transgender, only a subset will identify themselves within primary care—eg, individuals with emotional distress who seek support, those requesting gender affirming treatment, or those who otherwise believe their primary care physicians should know that they are transgender. A subset of this group will be referred on for specialist gender care. The proportion of people identified as having transgender identity is therefore likely to be higher in primary care samples than in secondary care samples, but lower than the proportion of people identifying as transgender in the general population.

Understanding the size and distribution of the transgender population is critically important for service design, resource allocation, and staff training, but estimating these factors is challenging,^15^ and high quality data are scarce.^16^ In 1998, Wilson and colleagues, using a cross-sectional survey of general practitioners in Scotland, estimated the proportion of people with gender dysphoria among those older than 15 years to be 0.82 per 10 000 individuals.^17^

No large scale, nationally representative, longitudinal studies have estimated the rates and proportion of transgender adolescents and adults identified within the UK's national public primary care system. We therefore aimed to report the proportion and change over time in rates of transgender people who presented to health care services based on UK primary care records.

## Method

### Study design

This cohort study was population based and used data from IQVIA Medical Research Data (IMRD), a proprietary database of anonymised clinical record data. IMRD incorporates data from The Health Improvement Network, a Cegedim database. The cohort study was dynamic; participants could enter and leave the study throughout the time period of interest.

Use of the terms incidence and prevalence have been criticised in the context of transgender health: firstly, for pathologising trans identity^18 19^; and secondly, for implying that transgender identity has an easily identifiable time of onset.^15^ In this article, we use the terms rate of first recording and proportion identified as transgender instead. From a statistical perspective, these figures are calculated in the same way as incidence and prevalence, and the rate ratios for newly recorded transgender codes and proportion identified as transgender are equivalent to incidence rate ratios and prevalence rate ratios.

### Setting

In the UK, almost all of the population are registered with an NHS primary care practice.^20^ Access is free of charge. The database IMRD contains de-identified data drawn from routinely collected primary care records. Approximately 6% of the UK's population are included in IMRD. This database is generally representative of the UK primary care population.^21^ Individuals can be registered at only one NHS primary care practice at a time: registering with a new practice triggers de-registration from the old practice. Diagnoses and observations are coded using the Read system, which is a hierarchical coding system including both diagnosis and symptoms.^22^ Social deprivation is estimated using the Townsend score, a combined measure of unemployment, car ownership, home ownership, and household overcrowding.^23^ Scores are defined for areas of approximately 150 households and grouped into fifths. The least deprived areas are slightly over-represented in IMRD, and the most deprived areas slightly under-represented: in 2009, 23.5% of patients active in the database belonged to the least deprived fifth, and 14.6% to the most deprived fifth.^21^

Adjusting for deprivation status in the data analysis is therefore important for its generalisability to the UK population. Townsend scores are calculated based on 2001 UK census data; people living in residential areas built after 2001, therefore, do not have data for deprivation in this dataset. We opted to use a complete case analysis, excluding individuals and practices with missing data for deprivation; use of a missing indicator method was likely to bias any analysis investigating a linear trend across the five groups.^24^

### Study population

We included data for individuals from 649 practices, excluding practices with missing data on Townsend deprivation (138 practices), and seven practices that supplied less than full calendar year of data. Roughly 8200 NHS general practitioner (GP) practices were recorded in the entire UK in 2021, although this number has been falling as smaller practices have tended to close over time.^25–28^

To determine the rate of first recording of transgender identity over time, we included all individuals aged 10-99 years who were permanently registered at a participating GP practice between 1 January 2000 and 31 December 2018; 2018 were the most recent data available at the time of the study. Individuals were included if they did not have a code suggestive of transgender identity on their records prior to, or within three months of, their registration at the practice (codes within three months were more likely to reflect re-coding of transgender identity already recorded at the individual's previous practice, based on visual inspection of Lewis plots^29^).

To determine the proportion of individuals with transgender identity over time, we included all individuals aged 10-99 years who were permanently registered at a participating practice and provided at least one full calendar year of data between 1 January 2000 and 31 December 2018. We considered individuals to be transgender from the date that the first qualifying code was recorded from then onwards.

### Definition of main outcome

A list of Read codes indicating transgender identity was developed using established methods^30^ and is presented in the online supplemental table 1. The presence of any of these codes in individuals' medical records was the main outcome measure.

10.1136/bmjmed-2023-000499.supp1 Supplementary data

### Identifying direction of transition

We sought to identify the direction of transition of individuals (ie, transmasculine—assigned female at birth and identifying as male, and transfeminine—assigned male at birth and identifying as female).

The database, IMRD, contains a variable for gender, coded as male or female. Whether this code refers to sex assigned at birth or current gender is not possible to determine; individuals can request to change their gender in primary care records at any time, and without any other requirements (such as after obtaining a gender recognition certificate, or having seen a specialist gender clinic).^31^ NHS numbers (the unique patient identifier given to every permanently registered NHS patient at a practice), and the unique identifier by which individual patient records are tracked within practices in the database, can only be male or female and this gender categorisation cannot change. A patient wishing to change their gender is, therefore, de-registered under the old NHS number, and re-registered under a new NHS number. Information from the old record should be transferred into the new one. In IMRD, this process would appear as a patient of one gender deregistering and a new one of another gender registering a similar time. However, distinguishing this change from two different people of different genders leaving and joining would be very difficult, particularly as patient level data in the database are pseudonymised (eg, birth dates are given by year only).

We opted instead to attempt to identify the direction of transition by examining related diagnostic codes and prescribed medications. Lists of examination findings and procedures suggesting patients who were assigned male at birth (eg, orchidectomy or construction of vagina) or assigned female at birth (eg, total abdominal hysterectomy or cervical screening normal) were constructed. These codelists are presented in the online supplemental tables 2 and 3. Lists of masculinising (ie, testosterone) and feminising medications (ie, oestrogens, GnRH (gonadotropin hormone-releasing hormone analogues, finasteride, spironolactone, dutasteride, and cyproterone) were also developed. Oestrogens and cyproterone were only included as sole ingredient formulations, that is, combined oral contraceptives containing oestrogens and progestogens were not included.

Among individuals with a transgender code (as defined above), presence of an assigned male at birth suggesting code or any prescription of feminising medication, or both, was taken to indicate transfeminine status. Presence of a code suggesting assigned female at birth or any prescription of masculinising medication, or both, was taken to indicate transmasculine status. DGJM individually reviewed and adjudicated for conflicted data (eg, individuals with a record containing both assigned male at birth and assigned female at birth codes).

### Statistical analysis

Analyses were stratified by age group, Townsend deprivation score, and calendar year.

The rate of new recording of transgender identity was estimated per 100 000 person years as the total number of people with newly recorded transgender identity between 2000 and 2018, divided by the total number of person years of follow-up. Person time was calculated as the time between the latest of: 10th birthday; three months after registration at the practice; 1 January 2000; acceptable computer usage date; acceptable mortality recording date (the latter two are quality assurance measures^32 33^), and the earliest of: date of first recorded transgender read codes; date of death; date of leaving the practice; date of last data collection from the practice; 99th birthday; 31 December 2018.

The proportion of people with transgender Read codes was determined by dividing the total number of people with a qualifying code by the total number of individuals in the eligible cohort (ie, all people aged 10-99, registered for at least one full calendar year), over the time period of interest (eg, the entire period, or for each calendar year when calculating proportion by year). Individuals could contribute data for more than one calendar year.

Poisson regression was done to obtain confidence intervals for the rate of newly recorded transgender identities and the proportion of people with transgender identity.

Multivariable Poisson regression models, with (log) person time as an offset, were used to determine rate ratios for newly recorded transgender status and risk ratios for proportions, adjusting for age, Townsend deprivation scores, and calendar year. Multilevel random intercept models were used to adjust for the effect of clustering by GP practice. Wald tests were used to examine for evidence of a linear relation between increasing Townsend deprivation score and transgender identity recording or proportion. Nested models, incorporating an interaction term, and a likelihood ratio test were used to examine for an interaction between age (dichotomised as <18 years and ≥18 years) and socioeconomic deprivation.

Visual inspection of the association between calendar year, rate of new recording, and proportion of people with trans identity, showed that rates and proportions were similar between age groups from 2000 to 2009, but diverged hugely beyond that point. Adding an interaction term ((2009 or earlier/2010 or later)*(age group)) to the multivariable modelling showed a highly significant interaction (P<0.0001)—that is, the association between new trans recordings or trans proportions and age group differed between 2000 and 2009 and 2010 and 2018. Rates of new recordings or proportions by age group were therefore reported separately for those two time periods.

Statistical analysis was performed using Stata 17.0 (StataCorp, College Station, TX, USA).

### Sensitivity analyses

To investigate the effect of excluding individuals and practices with missing Townsend data, we performed a sensitivity analysis to compare the point estimates obtained for rate of recoding and proportion with the full data set, versus the complete case analysis used above. The point estimates did not substantially differ. We therefore opted to retain the complete case analysis method; not adjusting for deprivation status leads to a slight bias towards least deprived areas, which are over-represented in the database.

### Patient and public involvement

Transgender and non-binary people were involved in the conduct of this research. One coauthor is non-binary, and another is transgender and has personal experience of seeking and receiving gender affirming care in the UK. Both coauthors were involved from the stage of interpretation and reporting of the findings, including the writing and critical revision of this paper, and have advised on appropriate methods of dissemination of the findings.

The research findings cannot be sent directly to the participants in the research because they are not individually identifiable by the researchers. The results will be disseminated to transgender and non-binary people through traditional media, social media, and communication with organisations supporting trans and non-binary people.

## Results

The absolute number of people identified as transgender in primary care medical records was small. Of 7 064 829 individuals aged 10-99 years, contributing at least a year of data between 2000 and 2018, 2462 (0.03%) had a Read code indicating transgender identity. Between 2000 and 2018, the overall rate of first recording of transgender identity was 2.2 per 100 000 person years (95% confidence interval 2.1 to 2.3), and the overall proportion of people who were transgender was 1.8 (95% confidence interval 1.8 to 1.9) per 10 000.

### Transmasculine and transfeminine status

Of the 2462 individuals with transgender identity, 417 (17%) appeared to be transmasculine (ie, prescribed testosterone, or had diagnostic or procedure codes suggesting that they were assigned female at birth, or both), and 923 (37%) appeared to be transfeminine (ie, prescribed feminising medication, or had diagnostic or procedure codes suggesting that they were assigned male at birth, or both). The remaining 1122 (46%) individuals could not be categorised: 1096 (45%) had no relevant codes or prescriptions to indicate the direction of transition, and 26 (1%) had conflicting data that could not be reconciled on manual review (eg, prescriptions for both masculinising and feminising medications).

Owing to the high rate of missing data for transmasculine and transfeminine status, we opted not to stratify by this variable in further analyses.

### Changes over time

**Figure 1:**
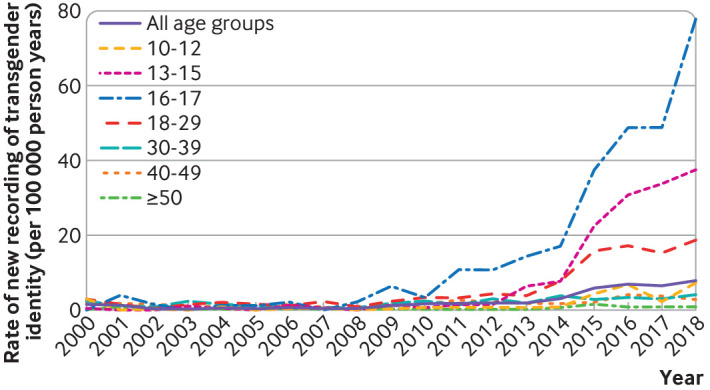
Rates of newly recorded transgender identity during 2000-18, by age group

The rate of first recordings of transgender identity increased substantially for all age groups between 2000 and 2018 (figure 1; overall, from 1.45 (95% confidence interval 0.96 to 2.10) per 100 000 person years to 7.81 (6.57 to 9.22) per 100 000 person years). The greatest proportional increase from 2000 to 2018 was in the 16-17 years age group, where the rate of first recording increased from zero (0 to 9.04) and 4.01 (0.49 to 14.47) per 100 000 person years, to 78.39 (54.60 to 109.02) per 100 000 person years. The rates of first recording appeared to markedly increase in the 10-12, 13-15, 16-17, and 18-29 age groups from about 2013-14 onwards (figure 1).

**Figure 2:**
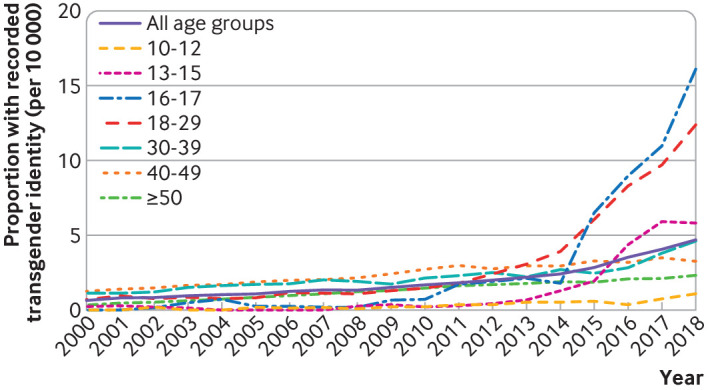
Proportion of people with recorded transgender identity between 2000 and 2018, by age group

Likewise, the proportion of people with a recorded transgender identity in their records increased for all age groups over time (figure 2) (overall, from 0.68 (95% confidence interval 0.55 to 0.83) per 10 000 in 2000, to 4.71 (4.38 to 5.05) per 10 000 in 2018). Again, individuals aged 16-17 years showed the greatest proportionate increase, from 0.19 (0 to 1.07) per 10 000 in 2002 to 16.23 (12.60 to 20.57) per 10 000 in 2018, with a sharp increase from 2015 onwards. Similar changes in the rate of increase were noted in the 18-29 years age group and in the 13-15 years group. In 2018, the proportion of people with transgender identity codes had reached 16.23 per 10 000 (12.60 to 20.57) in the 16-17 years group and 12.42 per 10 000 (11.06 to 13.90) in the 18-29 years groups. The proportion of people with recorded transgender identity in the 30-39, 40-49, and 50 years and older groups increased over time in a more gradual way.

The full data on which figures 1 and 2 are based on are given in online supplemental tables 4 & 5.

### Age and deprivation

Overall, the rate of first recordings of transgender identity was highest in the 16-17 years age group (12.8 new recordings per 100 000 person years (95% confidence interval 11.0 to 14.7)). The overall proportion of people with recorded transgender identity was highest in the 16-17 (2.31 per 10 000 (95% confidence interval 2.06 to 2.59)) and 18-29 age groups (2.67 (2.56 to 2.79)). Table 1 contains the rates of first recordings and proportions per age group between 2010 and 2018, and online supplemental table 6 contains the rates and proportions per age group between 2000 and 2009.

**Table 1:** Rates of new recordings and proportions of transgender identity per age group (2010-18) and Townsend deprivation score group (2000-18)

	Rate of newly recorded codes, per 100 000 person years (95% CI)	Rate ratio of newly recorded codes* (95% CI)	Proportion of people with transgender identity, per 10 000 (95% CI)	Rate ratio of proportion with transgender identity* (95% CI)
Age group (years):				
10-12	2.16 (1.37 to 3.25)	0.08 (0.05 to 0.14)	0.49 (0.33 to 0.69)	0.11 (0.08 to 0.16)
13-15	12.26 (10.24 to 14.57)	0.50 (0.40 to 0.63)	1.76 (1.51 to 2.05)	0.40 (0.33 to 0.48)
16-17	24.51 (20.95 to 28.50)	1 (ref)	4.44 (3.94 to 4.99)	1 (ref)
18-29	8.36 (7.54 to 9.25)	0.34 (0.28 to 0.40)	4.48 (4.27 to 4.69)	0.96 (0.85 to 1.09)
30-39	2.71 (2.23 to 3.26)	0.11 (0.09 to 0.14)	2.67 (2.50 to 2.84)	0.56 (0.49 to 0.64)
40-49	2.33 (1.91 to 2.80)	0.10 (0.08 to 0.12)	2.99 (2.83 to 3.16)	0.68 (0.60 to 0.77)
≥50	0.65 (0.51 to 0.81)	0.03 (0.02 to 0.04)	1.81 (1.73 to 1.89)	0.42 (0.37 to 0.47)
Townsend deprivation grouping:				
First (least deprived)	1.47 (1.28 to 1.67)	1 (ref)	1.16 (1.10 to 1.21)	1 (ref)
Second	1.67 (1.46 to 1.90)	1.11 (0.92 to 1.34)	1.42 (1.35 to 1.49)	1.25 (1.17 to 1.35)
Third	2.39 (2.13 to 2.68)	1.43 (1.20 to 1.71)	1.98 (1.90 to 2.07)	1.65 (1.54 to 1.77)
Fourth	2.77 (2.47 to 3.10)	1.55 (1.30 to 1.85)	2.07 (1.98 to 2.16)	1.66 (1.55 to 1.79)
Fifth (most deprived)	3.13 (2.75 to 3.56)	1.59 (1.31 to 1.92)	3.23 (3.09 to 3.37)	2.45 (2.28 to 2.65)

CI=confidence interval.

*Adjusted for age group, Townsend deprivation score, and calendar year.

**Figure 3:**
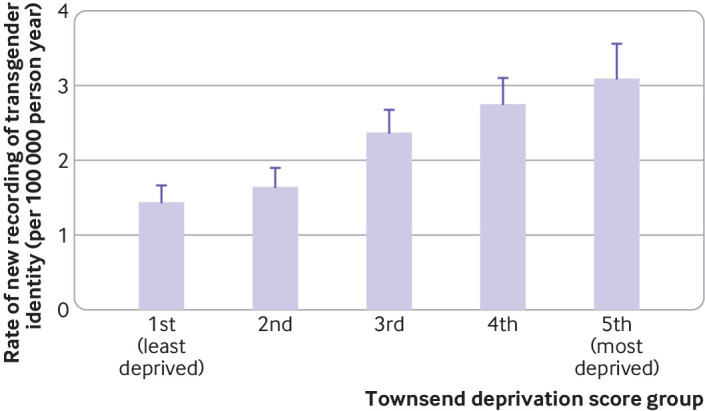
Rate of newly recorded transgender identity, per Townsend deprivation score group

**Figure 4:**
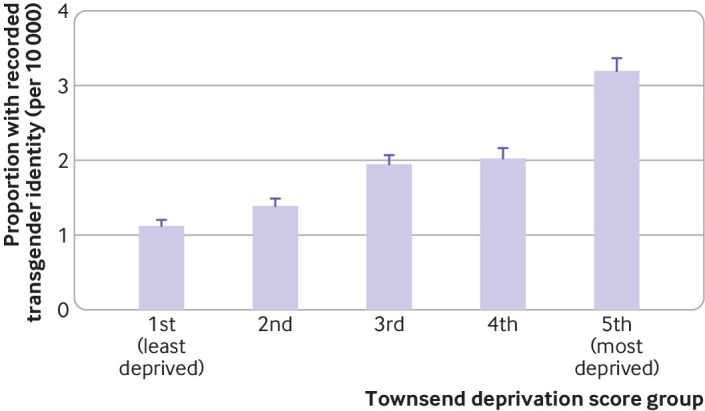
Proportion of people with recorded transgender identity, per Townsend deprivation score group

The rate of recording of new codes and the proportion of people with transgender identity showed a clear association with deprivation. People in the most deprived areas were 59% more likely to have a recorded transgender identity than people in the least deprived areas (adjusted rate ratio for new recording 1.59 (95% confidence interval 1.31 to 1.92), P for trend<0.0001). The association was stronger for the proportions of transgender identity, with individuals in the most deprived area being more than twice as likely to have a recorded transgender identity than individuals in the least deprived areas (adjusted rate ratio for proportion with transgender identity 2.45 (95% confidence interval 2.28 to 2.65), P for trend<0.0001) (table 1, figures 3 and 4). Socioeconomic deprivation and age showed no evidence of interaction (P=0.69 for likelihood ratio test).

## Discussion

### Principal findings

We report the first estimates of the rate of first recording and proportion of young people and adults with transgender medical record codes in a large primary care database that is nationally representative of the UK. The absolute number of individuals with codes related to transgender was small (0.03%). Transgender identity was most likely to be recorded for the first time in 16-17 year olds, and the proportion of people with recorded transgender identity was greatest between the ages of 16 and 29 years. The rates of newly recorded transgender identity and the proportion of people with a transgender code have increased markedly in all age groups between 2000 and 2018. A clear association between socioeconomic deprivation and recorded transgender identity was shown.

### Strengths and limitations

This study uses a very large dataset of real-world primary care records, from a sample that is broadly representative of the UK primary care population, to provide estimates with high external validity.

The main limitation of this study is the reliance on coding of transgender identity in clinical records. To receive a code that indicates a transgender identity, a transgender person would need to disclose their transgender identity to their primary care physician, the clinician would need to consider this relevant information to include in the clinical notes, and also they would need to code as such using one of the specified read codes.

The Read codes available do not fully capture the range of gender identity, such as non-binary or gender diverse identities, and contain various terms that are now outdated, or misapplied (eg, transsexuality). A time lag might also happen between initial presentation to primary care and coding of transgender identity, if, for example, the code is only applied after review by a gender identity clinic (for which the waiting time may be years). Our study cannot capture information from transgender people who do not share this information with their GP practice (where stigma may preclude disclosure^34^), and will therefore underestimate the true proportion of people with transgender identities in the population. Our study estimates the proportion of people for whom primary care clinicians are aware of their transgender status. Transgender individuals might also be receiving gender care solely in the private sector, or be self-medicating with hormone therapy, which primary care clinicians may not be aware of.^16^

We were only able to characterise the direction of transition (transmasculine or transfeminine) in just over half of transgender people. Data for the direction of transition should ideally be collected prospectively (eg, by asking individuals their sex assigned at birth and current gender identity^35^), but future work might examine alternative approaches for classifying transmasculine and transfeminine individuals, such as the use of free text searches. Further work should also attempt to collect data for non-binary and gender diverse individuals.

Ethnic group is an important intersectional factor; transgender people from ethnic minority backgrounds experience additional discrimination.^36^ Sexual orientation is distinct from gender identity, but sexuality and gender identity can change and interact in complex ways throughout life.^37^ Although ethnic group and sexual orientation may be recorded for some individuals in primary care, they are not routinely recorded and, therefore, we did not include these characteristics in our study.

The most recent data presented here is from 2018; recording rates of transgender identity in primary care have likely changed in the years since. If the trends in our study have remained, these rates have likely continued to increase; however, future work should extend this study to later timepoints.

Our study assumed that individuals retained a transgender identity after having any diagnostic code added; determining whether codes were later modified or removed was not possible. Gender identity is fluid and some people will choose not to transition to a different gender, and others may detransition.^38^ Our estimates therefore may include people who did not retain a transgender identity.

### Comparison with existing literature

Our overall proportion estimate for transgender identity of 1.8 per 10 000 is similar to those reported by studies based on the US healthcare system.^15 39–43^ Likewise, our earliest proportion estimate of 0.68 per 10 000 (95% confidence interval 0.55 to 0.83) in 2000 is similar to that of Wilson and colleagues’ estimate of 0.82 per 10 000 in 1998.^17^ Our findings of substantial increases in both the rate of first recording and the proportion of people with transgender identity (in all age groups) is consistent with similar studies in US healthcare system.^15^ Data from the UK also indicate increasing referral rates to gender identity clinics,^5 44^ and increases in applications for gender recognition certificates.^45^

Our method of identifying transgender individuals was based on diagnostic record codes. Other methods used elsewhere are of self-identified gender status, which is a more valid measure of the construct of transgender identity that is fundamentally self-defined.^46^ However, doing so requires prospective recording of self-defined gender status, which is not present in most electronic health records,^47^ including the one on which this study is based. Free-text searches of uncoded clinical records can also identify additional transgender individuals.^48^ Free text data, however, is variably available in research databases, and some ethical and privacy concerns surround making these data more accessible for analysis.^49^

Increasing rates of transgender codes in records may represent increasing numbers of people presenting to primary care with gender related concerns. Reasons for such may include increased availability of information, support and resources online, and increased societal awareness and acceptance, all of which have partially destigmatised transgender identities and may make coming out as transgender easier for individuals.^15^ These increases may also have affected by individuals' self-labelling, for example, leading them to conceptualise gender dysphoria and distress as an expression of a transgender identity. Changes in transgender identity recording may also represent improved recognition, knowledge, support, and coding by primary care clinicians.

The UK's Office for National Statistics has just begun to release estimates on the size of the transgender population in England and Wales, after a question about gender identity was asked for the first time in the 2021 Census. The Office for National Statistics data will be gradually released throughout 2023, but an initial report indicates that 0.5% of respondents felt that their gender identity did not match their sex registered at birth; the proportion of respondents specifically identifying as trans male or trans female was 0.1% in both cases.^50^ The 0.5% estimate is three times higher than our largest (and most recent) estimate of proportion, namely, 0.16% among 16-17 year olds in 2018. This is consistent with the literature, where survey based estimates of transgender identity are substantially higher than those based on medical record studies.^15^ Some people with transgender and gender questioning identities likely do not feel the need to seek medical treatment related to gender, or do not wish for their gender to be added to their medical record. However, if more people, whose gender care needs are hitherto unknown, do come forward to seek gender treatment in future, which seems probable, then the number of transgender people identified within primary care will continue to increase.

We found an association between socioeconomic deprivation, rates of newly recorded transgender identity, and the proportion of people with transgender identity. Clinic based samples of transgender people receiving specialist care have long reported disproportionately high levels of deprivation,^51 52^ along with stigma and isolation from family members, difficulties securing employment, and challenges in personal relationships.

Although evidence is clear that transgender people face discrimination in many forms,^13^ few population based studies have assessed the association between transgender status and socioeconomic deprivation.^53^ Two recent studies, one in Denmark,^54^ and one in the US,^53^ reported lower household incomes and lower employment rates in transgender people compared with a cisgender comparison group.

The direction of causality between socioeconomic status and transgender status is challenging to determine, and we cannot confidently explain the association from our data. Transgender individuals in wealthier areas may be more able to afford specialist gender care privately, which can be accessed entirely independently of NHS primary care. This trend may be increasing with longer NHS waiting lists. Therefore, individuals from a wealthier background might bypass NHS services entirely. However, primary care might not be entirely unaware of registered patients who are having gender affirming care in the private sector, especially as shared care requests from the private sector are common—enough so that regional and national NHS bodies have produced guidance on how to handle such requests.^4 11^

Transgender adults might face discrimination and stigma leading to exclusion from education, employment, and family support, and therefore become more likely to move to socioeconomically deprived areas. Potentially, these areas might also be more accepting of transgender individuals than others. Transgender populations have higher rates of substance abuse and mental illness compared with cisgender peers,^55^ which may be partially explained by the minority stress model^56^; therefore, the burden of these health issues might affect income, employment, and societal integration. However, this explanation would not account for the association between transgender identity and deprivation seen in young people, whose socioeconomic status is largely determined by their parents' wealth, and therefore predates the development of transgender identity. Higher parental wealth may allow young people to access care privately, independently of their NHS GP.

## Conclusions

We have presented data for the rate of first recording, and the proportion of people with diagnostic codes for transgender identity in UK primary care records between 2000 and 2018. Although the absolute proportion of people identified as transgender in primary care records is low, in relative terms, the number increased substantially over this period. As such, resources must be allocated to primary and specialist care to meet the healthcare needs of these individuals. Socioeconomic deprivation in both adults and children is associated with a greater proportion of people with codes suggesting transgender identity; the reasons for this association are unclear, and should be explored in future research.

## Data Availability

Data may be obtained from a third party and are not publicly available. The data used in this study are available from IQVIA Medical Research Data. Restrictions apply to the availability of these data, which were used under licence for this study.
