# Supplementary material for: Transgender identity in young people and adults recorded in UK primary care electronic patient records: retrospective, dynamic, cohort study
Source: BMJ Med. 2023 Nov 28;2(1):e000499. doi: 10.1136/bmjmed-2023-000499 (PMC10685922; doi:10.1136/bmjmed-2023-000499)
Supplement: Supplementary data [file bmjmed-2023-000499supp001.pdf]

Supplementary table 1: list of Read codes taken to indicate transgender identity, and number of individuals with that code.

Read code	Description	Number of individuals with code recorded
Eu64.00	[X]Gender identity disorders	1481
E22y400	Gender role disorder of adolescent or adult	357
1K4..00	Gender reassignment patient	354
Eu64000	[X]Transsexualism	290
ZV62A00	[V] Gender dysphoria	244
Eu64z11	[X]Gender-role disorder NOS	141
7L0B.00	Operations for sexual transformation	115
Eu64z00	[X]Gender identity disorder, unspecified	93
7L0B000	Combined operations for transformation from male to female	55
7L0Bz00	Operation for sexual transformation NOS	10
Eu64y00	[X]Other gender identity disorders	9
Eu64200	[X]Gender identity disorder of childhood	9
7L0B100	Combined operations for transformation from female to male	8
E225z00	Trans-sexualism NOS	6
E225200	Trans-sexuality with homosexual history	≤5
E225300	Trans-sexuality with heterosexual history	≤5
E225000	Trans-sexuality with unspecified sexual history	≤5
E225100	Trans-sexuality with asexual history	≤5
7L0By00	Other specified operation for sexual transformation	≤5

To preserve confidentiality, absolute counts of five or fewer individuals are suppressed.

McKechnie DGJ, *et al. BMJMED* 2023; 2:e000499. doi: 10.1136/bmjmed-2023-000499

Supplementary table 2: list of Read codes taken to indicate patient was likely assigned male at birth.

B483.00	Malignant neoplasm of penis, part unspecified	7B36z11	Prostatectomy NEC
7B3BC00	Endoscopic radiofrequency ablation of lesion of prostate	K27y300	Fibrosis of penis
43ZB000	Serum free prostate specific antigen level	7C20000	Total amputation of penis
7B36700	Radical prostatectomy with pelvic lymphadenectomy	7B3B.11	Trans-urethral microwave thermotherapy to prostate.
7B2AE00	Diag endoscop examination bladder biopsy lesion prostate NEC	PCyy.00	Other congenital anomaly of penis
9OkD000	Prostate specific antigen monitoring first letter	K27y000	Oedema of penis
7B3Cy00	Other operation on outlet of male bladder or prostate OS	43ZB.00	Free prostate specific antigen level
7C05100	Other one stage bilateral orchidopexy	B481.00	Malignant neoplasm of glans penis
7B3C.00	Other operations on prostate or male bladder outlet	7C2..00	Penis and other male genital organ operations
7B36600	Radical prostatectomy with pelvic node sampling	4M02.00	Gleason prostate grade 8-10 (high)
7C25y00	Other specified other operation on penis	PC50000	Cryptorchidism, unilateral
7B37400	Open haemostasis of prostate	25QB.00	On rectal exam of prostate NAD
8Cd6.00	Advice given about prostate cancer screening	ZV76800	[V]Special screening examination for neoplasm of prostate
PC5..00	Undescended testicle	B7C1.00	Benign neoplasm of penis
7C21300	Cryotherapy to lesion of penis	K272.11	Infection of penis
7B20200	Radical cystoprostatectomy	7C22900	Graft to penis
7B3CA00	Transurethral radiofrequency needle ablation of prostate	7C06000	Unilateral microvascular orchidopexy
7B39311	ELAP - Endoscopic laser ablation of prostate	SD11A00	Abrasion of penis, infected
7C02000	Bilateral subcapsular orchidectomy	7B36111	Millin retropubic prostatectomy
B916000	Neoplasm of uncertain behaviour of penis	8A90.00	Prostate-specific antigen monitoring
7C02211	Bilateral total inguinal orchidectomy	85C3.00	Inject prostate - local action
PCyy100	Webbed penis	7B39500	Endoscopic resection of prostate using an electrotome
7B3B911	Vapatrode prostatectomy	K27y400	Hypertrophy of penis
SD15A00	Insect bite, nonvenomous, of penis, infected	7B37y00	Other specified other open operation on prostate
7B39100	Punch resection of prostate	SG93.00	Foreign body in penis
7C25D00	Removal of constricting object from penis	7D16012	Williams construction of vagina

7B37100	Open biopsy of prostate	7B3Cz00	Other operation on outlet of male bladder or prostate NOS
K22y200	Stricture of prostate	7B3E000	High intensity focused ultrasound of prostate
7C02100	Bilateral total orchidectomy, unspecified method	7C05.00	Bilateral orchidopexy
S880.00	Open wound of penis	7B3BB00	Endoscopic microwave destruction of lesion of prostate
7C23.00	Prosthesis of penis	7B39.00	Endoscopic resection of outlet of male bladder or prostate
9N1l.00	Seen in prostate clinic	ZV10415	[V]Personal history of malignant neoplasm of prostate
7C22300	Frenuloplasty of penis	7B3Ez00	Other operations on prostate NOS
85C3.11	Prostate injection	5B51.00	Microwave therapy to prostate
25Q2.11	Prostate enlarged on PR	7C03400	Unilateral scrotal orchidectomy
7C21200	Other destruction of lesion of penis	S880000	Degloving injury penis
PCy1400	Aplasia of penis	PCyy000	Hooded penis
SD14A00	Insect bite, nonvenomous, of penis	7C06y00	Other specified unilateral orchidopexy
7B3By00	Other therapeutic endoscopic operation on prostate OS	SD13A00	Blister of penis, infected
7C25.00	Other operations on penis	SD17A00	Splinter of penis without major open wound, infected
7B3B900	Endoscopic transurethral electrovaporisation of prostate	1J08.00	Suspected prostate cancer
7B39.12	Endoscopic prostatectomy	7B36100	Retropubic prostatectomy
7C03200	Unilateral total orchidectomy - unspecified	K20..14	Enlarged prostate - benign
7C21.00	Extirpation of lesion of penis	7B36300	Perineal prostatectomy
7C03211	Orchidectomy NEC	7B39000	Transurethral prostatectomy
7C05111	Bevan bilateral orchidopexy	K224.00	Amyloid of prostate
7B3B100	Other endoscopic destruction of lesion of prostate	7B36.00	Open prostatectomy
66Z0.00	Prostate specific antigen threshold for referral	Kyu6400	[X]Other specified disorders of penis
4M01.00	Gleason prostate grade 5-7 (medium)	7C03000	Unilateral subcapsular orchidectomy
25QA.00	O/E - PR - prostate median sulcus preserved	7B3z.00	Bladder neck and prostate operations NOS
K223.00	Dysplasia of prostate	B46..00	Malignant neoplasm of prostate
B7C2200	Myoma of prostate	A541300	Herpetic infection of penis
8AD0.00	Active surveillance of prostate cancer	K220.11	Stone of prostate
K22y.00	Other disorders of prostate OS	7C22.00	Plastic operations on penis
7B3C800	Transrectal microwave thermotherapy to prostate	PCy1000	Congenital aplasia of prostate

7B3E100	Implantation of radioactive substance into prostate	PC50.00	Cryptorchidism
7C21z00	Extirpation of lesion of penis NOS	7B3C500	Transurethral biopsy prostate
7C02300	Bilateral scrotal orchidectomy	26J0.11	O/E - Clapper-bell testicle
B7C1100	Benign neoplasm of glans penis	PCy0100	Congenital absence of prostate
K21..11	Prostatitis and other inflammatory diseases of prostate	PCy7.00	Congenital lateral curvature of penis
K22y100	Infarction of prostate	K27..00	Disorders of penis
7C2..12	Penis operations	7C2y.00	Other specified operations on penis or other male organ
7C21100	Cauterisation of lesion of penis	7B36500	Radical prostatectomy without pelvic node excision
K27y200	Atrophy of penis	K20..13	Benign myoma of prostate
K274.12	Induratio penis plastica	7B3Dy00	OS endoscopic insertion of prosthesis into prostate
7B3BA00	Endoscopic transurethral microwave thermotherapy to prostate	43Z2000	Prostate specific antigen normal
SD10A00	Abrasion, penis	7C02200	Bilateral inguinal orchidectomy
K20..11	Benign adenoma of prostate	K21..00	Prostate inflammatory diseases
14N8.00	H/O: orchidectomy	K270.12	Leucoplakia of penis
7B3E.00	Other operations on prostate	K275.00	Vascular disorders of penis
7B2A100	Unspec diagnostic cystoscopic exam bladder & biopsy prostate	B7C2.00	Benign neoplasm of prostate
Kyu6800	[X]Disorders of prostate in diseases classified elsewhere	7B39300	Endoscopic laser ablation of prostate
K22yz00	Other prostate disorders NOS	B7C2100	Fibroma of prostate
43ZC000	Serum free:total prostate specific antigen ratio	7C03300	Unilateral inguinal orchidectomy
2661.00	O/E - penis normal	2656.00	O/E - testicles normal
9OkD100	Prostate specific antigen monitoring second letter	7B36.11	Open excision prostate
7B37.00	Other open operations on prostate	A165000	Tuberculosis of prostate
7B3Dz00	Endoscopic insertion of prosthesis into prostate NOS	7B36z00	Open excision of prostate NOS
26J..00	O/E - testicle	SD1yA00	Superficial injury of penis NOS, without major open wound
7B3C900	Seed implantation into prostate	7N51611	[SO]Skin of penis
1A5B.00	Pain in penis	Pyu6900	[X]Oth cong malform vas def/epidid/semin vesicles/prostate
Pyu6A00	[X]Other congenital malformations of penis	B915.00	Neoplasm of uncertain behaviour of prostate
7C25z00	Other operation on penis NOS	7B3B000	Endoscopic cryotherapy of prostate

PC5z.00	Undescended testicle NOS	7C21y00	Other specified extirpation of lesion of penis
B482.00	Malignant neoplasm of body of penis	ZX1L300	Self-mutilation of penis
PCy1200	Congenital aplasia of testicle	7C22z00	Plastic operation on penis NOS
7B36y00	Other specified open excision of prostate	7C06100	One stage unilateral orchidopexy
38GT700	QCancer prostate cancer risk	43Z2100	Prostate specific antigen abnormal
7B39800	Endoscopic excision of prostate using holmium laser	7C22y00	Other specified plastic operation on penis
7B3C400	Balloon dilation of prostate	SF10000	Crush injury, penis
7B39.13	Endoscopic resection of prostate	K270.11	Kraurosis of penis
S881.00	Open wound of penis with complication	Kyu6300	[X]Other inflammatory disorders of penis
7C02z00	Bilateral orchidectomy NOS	B7C1z00	Benign neoplasm of penis NOS
2658.00	O/E - testicles small	124H.00	Family history of prostate cancer
7C21400	Extracorporeal shockwave lithotripsy to lesion of penis	7C05112	Ombredanne bilateral orchidopexy
7D16000	Construction of vagina	9OkD.00	Prostate specific antigen monitoring invitation
7B3C100	Transperineal needle biopsy of prostate	7C2z.00	Penis and other male organ operations NOS
7C22200	Nesbitt's operation on penis	Kyu6100	[X]Other specified disorders of prostate
7B37z00	Other open operation on prostate NOS	Kyu6000	[X]Other inflammatory diseases of prostate
7C22211	Plication of corpora of penis	7B3B111	Endoscopic destruction of lesion of prostate NEC
7B36400	Transvesical two stage prostatectomy	7C25300	Suture of penis
7A54C00	PAE - prostate artery embolisation	B487.00	Malignant neoplasm, overlapping lesion of penis
4L4J.00	Urine prostate cancer antigen 3 targeted genetic test	A32y500	Diphtheria of penis
7B3CB00	Radioactive seed implantation into prostate	B48z.00	Malignant neoplasm of penis and other male genital organ NOS
7C05000	Bilateral microvascular orchidopexy	7B3y.00	Other specified operations on bladder neck or prostate
7B3..00	Bladder outlet and prostate operations	7C06.00	Unilateral orchidopexy
4M00.00	Gleason prostate grade 2-4 (low)	SE24100	Contusion, penis
7C20z00	Amputation of penis NOS	7C22800	Repair of fracture of penis
7C25200	Other incision of penis	B58y500	Secondary malignant neoplasm of prostate
7C25100	Drainage of penis-unspecified	7C05300	Second stage bilateral orchidopexy
9OkD200	Prostate specific antigen monitoring third letter	SD1zA00	Superficial injury of penis NOS, infected
7B3A400	Transurethral incision of prostate	7C06z00	Unilateral orchidopexy NOS
PCyx.00	Other congenital anomaly of vas deferens or prostate	K27y500	Chronic ulcer of penis

7B3C600	Cryotherapy to prostate via perineal probe	43Z2.11	PSA - Serum prostate specific antigen level
7C2..11	Penis & other male organ op	S77v200	Prostate injury without mention of open wound into cavity
8L51.00	Prostatectomy planned	7C06111	Ombredanne unilateral orchidopexy
7B39700	Endoscopic resection of prostate using vapotrode	K220.00	Calculus of prostate
7B3C111	Trucut transperineal biopsy of prostate	7B3B300	Endoscopic punch biopsy of prostate
7B3D.00	Endoscopic insertion of prosthesis into prostate	B7C1000	Benign neoplasm of prepuce of penis
7B3B.00	Other therapeutic endoscopic operations on prostate	7C06200	First stage unilateral orchidopexy
7C21000	Excision of lesion of penis	1427000	H/O: prostate cancer
7C27100	Dermatological non-operative interventions involving penis	7N51100	[SO]Prostate
PC5z.12	Maldescent of testicle	7B3C011	Franzen needle aspiratation of prostate
266..00	O/E - penis	2663.11	O/E - discharge - penis
SD12A00	Blister of penis	7N51600	[SO]Penis
S77w200	Prostate injury with open wound into cavity	K272300	Cellulitis of penis
8HTz.00	Referral to prostate assessment clinic	7C25F00	Operations on penis for erectile dysfunction NEC
K270.00	Leukoplakia of penis	PC50z00	Cryptorchidism NOS
43ZG.00	Ultra-sensitive prostate specific antigen level	25Q8.00	O/E - PR - prostate tender
7B3Ey00	Other specified other operations on prostate	B58y700	Secondary malignant neoplasm of penis
K22..00	Other disorders of prostate	PC50100	Cryptorchidism, bilateral
7B39200	Other endoscopic resection of prostate	7C02.00	Bilateral orchidectomy
7B3B012	Thermex thermotherapy of prostate	B7C2000	Adenoma of prostate
PCy2000	Hypoplasia of penis	7B3C200	Transrectal needle biopsy of prostate
7B3C700	Transurethral microwave thermotherapy to prostate	58D4.00	Transrectal ultrasound scan of prostate
7B3B600	Ultrasound ablation of prostate	4M0..00	Gleason grading of prostate cancer
7C20.00	Amputation of penis	43Z2200	Serum prostate specific antigen level
7C20100	Partial amputation of penis	7B3B011	Endoscopic cryotherapy to lesion of prostate
7B3C000	Needle aspiration of prostate	26J0.00	O/E - Bell-clapper deformity of testicle
B835.00	Carcinoma in situ of penis	7C02y00	Other specified bilateral orchidectomy
7C20y00	Other specified amputation of penis	K20..12	Benign fibroma of prostate
7B3B200	Endoscopic drainage of prostate	7C05200	First stage bilateral orchidopexy
7B36200	Transvesical prostatectomy	43ZC.00	Free:total prostate specific antigen ratio

7C02311	Bilateral total scrotal orchidectomy	7C25000	Biopsy of penis
25Q9.00	O/E - PR - Prostate median sulcus not preserved	G82z111	Penis vein thrombosis
B48..00	Malignant neoplasm of penis and other male genital organs	7D16011	McIndoe construction of vagina
B834.00	Carcinoma in situ of prostate		
7C23y00	Other specified prosthesis of penis		
266Z.00	O/E - penis NOS		
265C.00	Examination of testicle		
43Z2.00	Prostate specific antigen		
8BAV000	Prostate cancer care review		
7B3Bz00	Other therapeutic endoscopic operation on prostate NOS		
7B36411	Freyer two stage transvesical prostatectomy		
7B36000	Radical prostatectomy - unspecified excision of pelvic nodes		
SD16A00	Splinter of penis, without major open wound		
7C06300	Second stage unilateral orchidopexy		
7C25500	Injection of therapeutic substance into penis		
PCyyz00	Other congenital anomaly of penis NOS		
7B3C.11	Other operations on prostate		
7C05z11	Bilateral orchidopexy NOS		
K212.00	Abscess of prostate		
1A57.00	Pain in testicle		
388o.00	International prostate symptom score		
7B39600	Endoscopic resection of prostate using laser		
K222.00	Atrophy of prostate		

Supplementary table 3: list of Read codes taken to indicate patient was likely assigned female at birth.

L34y100	Other vulval/perineal trauma during delivery- delivered	K56y112	BPV - Vaginal bleeding
7E20z00	Partial excision of ovary NOS	2699.00	Uterine cervix transformation zone visualised
7D03311	Cauterisation of vulval warts	K53y200	Infarction of ovary
PC4y900	Congenital stenosis of vagina	K51y200	Relaxation of vaginal outlet
K56y000	Cyst of vagina	7D19z00	Repair of vault of vagina NOS
K587.00	Contact bleeding of cervix	269..00	O/E - vaginal speculum exam.
7D03400	Implantation of radioactive substance into vulva	7E05y00	Other specified vaginal excision of uterus
AB21111	Vaginal thrush	7E20200	Marsupialisation of lesion of ovary
7D14z00	Excision of band of vagina NOS	7E08000	Dilation cervix & vacuum aspirat products conception uterus
K42y500	Vulval vestibulitis	4K29100	Cervical smear - borderline change in endocervical cells
9kF7.00	Endocervical swab culture neg - enhanced services administra	R150.11	[D]Cervical smear - nonspecific abnormality
SH74000	Burn of the vagina	L247.00	Congenital/acquired abnormality vagina in preg/childb/puerp
7E2B.00	Other operations on ovary	K562z00	Stricture or atresia of the vagina NOS
7E16311	Open ringing of fallopian tube NEC	7N60z00	[SO]Vagina NEC
SD15D00	Insect bite, nonvenomous, of vagina, infected	PC4yz00	Other cervical/vaginal/external female genital anomaly NOS
L345100	Vulval and perineal haematoma during delivery - delivered	7E13.00	Partial excision of fallopian tube
PC1y200	Atresia of fallopian tube	K424000	Abscess of vulva
4K2..00	Cervical smear result	4K39.00	Cervical smear - gardnerella
7E16.00	Other open occlusion of fallopian tube	K401200	Chronic salpingo-oophoritis
Pyu6100	[X]Other congen malform of fallopian tube & broad ligament	7D17y00	Repair of vaginal prolapse & amputation of cervix uteri OS
PC4y700	Agenesis of vulva	7E04312	Hysterectomy NEC
4JKE.11	Self taken low vaginal swab	K53..00	Noninflammatory disorders of the ovary/tube/broad ligament
K564.00	Old vaginal laceration	PC4yw11	Vaginal septum
ZV13C00	[V]Personal history of moderate cervical dysplasia	K421600	Vulvovaginitis in diseases EC
7E00000	Amputation of cervix uteri	PCyB.00	Doubling of vagina
L411613	Vulval varices in the puerperium	PC4yB11	Imperforate vagina
L341.13	Vaginal muscle tear	L411200	VV's of perineum/vulva in pregnancy/puerperium -del+p/n comp
PC4yx00	Other congenital anomaly of vulva	M181.11	Pruritus vulvae

26A1.00	O/E - no vaginal discharge	7B32y00	Vaginal operation to support outlet of female bladder OS
SD16D00	Splinter of vagina, without major open wound	ByuFA00	[X]Carcinoma in situ of other parts of cervix
BBCE.00	[M]Lipid cell tumour of ovary	R150z00	[D]Nonspecific abnormal Papanicolaou cervical smear NOS
7E1Cz00	Endoscopic bilateral occlusion of fallopian tubes NOS	7E15000	Open bilateral ligation of fallopian tubes
7D15y00	Other specified extirpation of lesion of vagina	B412.00	Malignant neoplasm, overlapping lesion of cervix uteri
B44..00	Malignant neoplasm of ovary and other uterine adnexa	4JK3.00	Low vaginal swab taken
Kyu8300	[X]Other specified inflammation of vagina and vulva	7E05y11	Ward vaginal hysterectomy
4KK1.00	Vaginal vault smear repeat at 12 months	7D15.00	Extirpation of lesion of vagina
7E00y00	Other specified excision of cervix uteri	L246312	Stenosis of cervix complicating a/n care- baby not delivered
K288100	Tunica vaginalis stricture	K57y400	Lesion of vulva
7E22100	Suture of ovary	K516000	Congenital vaginal enterocele
4JK4.00	Vulval swab taken	685H.00	No smear - benign hysterectomy
SD10C00	Abrasion, vulva	K577000	Mild vulvar dysplasia
7D1y.00	Other specified operations on vagina	K570100	Leukoplakia of vulva
AB21000	Candidiasis of vulva	K520600	Vesicocervicovaginal fistula
7E04800	Abdominal hysterectomy and left salpingoophorectomy	7E02.00	Biopsy of cervix uteri
K287.00	Chylocele of tunica vaginalis	7N60y00	[SO]Specified vagina NEC
K40..00	Ovarian, fallopian tube and pelvic inflammatory diseases	7E18000	Reconstruction of fallopian tube
K532100	Theca lutein cyst of the ovary	K562100	Post-radiation vaginal adhesions
6853.00	Ca cervix screen - not wanted	7D1B.00	Introduction of supporting pessary into vagina
L247200	Vaginal abnormality - baby delivered+postpartum complication	PC4yv00	Other congenital anomaly of cervix
7B26100	Creation of vesicovaginal fistula	ZV10414	[V]Personal history of malignant neoplasm of ovary
Kyu9N00	[X]Dysplasia of vulva, unspecified	7D1A300	Repair of uterovaginal fistula
SD14D00	Insect bite, nonvenomous, of vagina	7E15y00	Other specified open bilateral occlusion of fallopian tubes
7N62200	[SO]Ovary	M181.12	Vulva sore
7D1..00	Vagina operations	Kyu9L00	[X]Severe vaginal dysplasia, not elsewhere classified
7D05200	Evacuation of haematoma of vulva	7E03311	Cerclage of cervix of non-gravid uterus
7E1Dy00	Other specified endoscopic occlusion of fallopian tube	B41y100	Malignant neoplasm of squamocolumnar junction of cervix
7E23.00	Other open operations on ovary	7E2..12	Ovary operations
AB21.00	Candidal vulvovaginitis	Kyu9M00	[X]Severe vulvar dysplasia, not elsewhere classified

AD10012	Leukorrhoea vaginalis - trichomonal	7E1z.00	Fallopian tube operations NOS
B833900	Vaginal intraepithelial neoplasia grade 3	A166.00	Tuberculous oophoritis or salpingitis
7E15z00	Open bilateral occlusion of fallopian tubes NOS	685..12	Cervical smear screen
1A5E.00	Pain in vulva	L411300	VV's of perineum/vulva in pregnancy/puerperium + a/n comp
K554100	Contracture of cervix	7E01100	Laser destruction of lesion of cervix uteri NEC
7E08500	Dilation of cervix and extraction termination of pregnancy	K402300	Oophoritis unspecified
7D05411	Separation of vulval adhesions	L340.15	Vulval tear
B790.11	Adenomatous polyp - cervix uteri	4K4C.00	Cervical smear repeat at 60 months
7D19700	Repair of vault of vagina with mesh using vaginal approach	7E03100	Dilation of cervix uteri
7E03000	Repair of cervix uteri NEC	L247411	Septate vagina complicating p/n care - baby delivered prev
7E21y00	Other specified open destruction of lesion of ovary	124C.00	FH: neoplasm of ovary
K53y.00	Other ovary, fallopian tube and broad ligament disorders	PC4z.00	Cervical, vaginal and external female genital anomaly NOS
7E19000	Removal of products of conception from fallopian tube	7E19011	Removal of ectopic pregnancy from fallopian tube
K533000	Acquired atrophy of the ovary unspecified	7D04300	Reconstruction of vulva with distant flap
K57yz00	Other noninflammatory vulval and perineal disorder NOS	L246.11	Polyp of cervix in pregnancy, childbirth and the puerperium
7E11400	Unilateral oophorectomy NEC	SD1zC00	Superficial injury of vulva NOS, infected
B833600	Vulval intraepithel neop grd 3	K514.00	Uterovaginal prolapse, unspecified
7E11200	Unilateral salpingectomy NEC	K551.12	CIN I - II, cervical dysplasia
7D11y00	Other specified other operation on introitus of vagina	7E16700	Open ligation of left fallopian tube
4K27.00	Cervical smear:atrophic change	4K3A.00	Cervical smear: koilocytosis
L248z00	Vulval abnormality in pregnancy/childbirth/puerperium NOS	K400z00	Acute salpingitis and oophoritis NOS
B831100	Carcinoma in situ of exocervix	7E2A.13	Other examination of vagina
4K2J.00	Cervical smear - low grade dyskaryosis	K402000	Fallopian tube abscess
7N61000	[SO]Cervix uteri	7E1D.11	Endoscopic unilateral occlusion of fallopian tubes
7E25400	Endoscopic extirpation of lesion of ovary NEC	7D1A400	Suture of vagina
7D15700	Excision of granulation tissue of vagina	K520300	Urethrovesicovaginal fistula
2689.00	O/E-VE-cervical excit.present	4K36.00	Cervical smear - wart virus
7E01.00	Destruction of lesion of cervix uteri	7D0..00	Vulva and female perineum operations
K573.11	Vulva hypertrophy NOS	K554011	Stenosis of cervix - acquired
7E25000	Endoscopic extirpation of lesion of ovary	4K2N.00	Cervical smear - ?endocervical type glandular neoplasia
9O8U.00	Cervical smear slide broken in transit	7E1B100	Open biopsy of fallopian tube

K57y100	Oedema of vulva	7E1B000	Open freeing of adhesions of fallopian tube
S77w000	Fallopian tube injury with open wound into cavity	K511.00	Uterine prolapse without vaginal wall prolapse
7E10100	Bilateral salpingectomy NEC	K516z00	Vaginal enterocele NOS
B833300	Carcinoma in situ of vulva	7E10200	Bilateral oophorectomy NEC
9O82.00	Cervical smear - 2nd call	K56y100	Haemorrhage of vagina
7E04G00	Total abdominal hysterectomy with conservation of ovaries	B410z00	Malignant neoplasm of endocervix NOS
7E05400	Laparoscopic vaginal hysterectomy	8C85.00	Gamete intrafallopian transfer
6855.00	Ca cervix screen - not needed	K554000	Acquired stricture of cervix
B7B1.00	Benign neoplasm of vagina	K510z00	Vaginal prolapse without uterine prolapse NOS
269Z.00	O/E -vaginal speculum exam.NOS	K510400	Vaginal prolapse unspecified without uterine prolapse
B913100	Neoplasm of uncertain behaviour of vulva	L247011	Septate vagina affecting obstetric care
4KK2.00	Vaginal vault smear repeat at 18 months	K575.00	Haematoma of vulva
142D.00	H/O: malignant neoplasm vulva	Z174D00	Saving clots passed via vagina
7D14y00	Other specified excision of band of vagina	L246212	Stenosis of cervix - baby delivered+postpartum complication
279Z.00	O/E - VE - cervix dilat. NOS	7D18600	Paravaginal repair
SD16C00	Splinter of vulva, without major open wound	7D11z00	Other operation on introitus of vagina NOS
7E2y.00	Other specified operations on ovary or broad ligament	7E10000	Bilateral salpingoophorectomy
7F06000	Cerclage of cervix of gravid uterus	6857.11	Cervical smear non-responder
B7B0000	Benign neoplasm of fallopian tube	B45y000	Malignant neoplasm of overlapping lesion of vulva
2781.00	O/E - VE - cervix not ripe	Kyu9D00	[X]Other specified abnormal uterine and vaginal bleeding
7E13z00	Partial excision of fallopian tube NOS	7E1D100	Endoscopic occlusion of right fallopian tube
L090400	Salpingo-oophoritis following abortive pregnancy	4KAZ.00	Vaginal vault smear NOS
7D1Dz00	Other operation on vagina NOS	7F1A000	Caesarean hysterectomy
1A72.00	Vaginal discharge present	7E04700	Abdominal hysterectomy and right salpingoopherectomy
7F18000	Manip cephalic vaginal deliv abnorm pres head without instrm	685M.00	Cervical smear overdue
7D1D600	Insertion of pack into vagina NEC	7B30y00	Combined abdo & vaginal op to support outlet fem bladder OS
ZV13B00	[V]Personal history of mild cervical dysplasia	L34y000	Other vulval/perineal trauma during delivery, unspecified
685R.00	Liquid based cervical cytology screening	K53yz00	Other ovary, fallopian tube and broad ligament disorder NOS
4JK2200	HVS culture - trichomonas vaginalis	7E00300	Endocervical excision NEC
124D.00	FH: neoplasm of cervix	K401100	Chronic perioophoritis
7D1z.00	Vagina operations NOS	7D05300	Sclerosing injection into vulval vein

158..12	Vaginal bleeding	KyuA100	[X]Residual ovary syndrome
7D15500	Excision of vaginal adhesions	K53y300	Rupture of ovary
7E11000	Unilateral salpingoophorectomy NEC	L247412	Stenosis of vagina complicating p/n care - baby deliv prev
7E04511	Abdominal hysterectomy & bilateral salpingoophorectomy (BSO)	7E05311	Heaney vaginal hysterectomy
K535100	Torsion of the ovary and fallopian tube	L411612	Vaginal varices in the puerperium
26AZ.00	O/E - vaginal discharge NOS	PC22.00	Doubling of uterus, including cervix and vagina
K534000	Prolapse of the ovary	K55y300	Haemorrhage of cervix
7E2A300	Vaginal vault smear	7E22.00	Repair of ovary
7E16z00	Other open occlusion of fallopian tube NOS	7F22700	Pack to control postnatal vaginal bleeding
L247112	Stenosis of vagina - baby delivered	7E04N00	Radical hysterectomy with conservation of ovaries
K551400	Moderate cervical dysplasia	7E2A.11	Other examination of cervix uteri
SD1yD00	Superficial injury of vagina NOS, without major open wound	7E17000	Reanastomosis of fallopian tube NEC
L248300	Vulval abn complicating a/n care - baby not yet delivered	SG92100	Foreign body in vagina
7E18300	Suture of fallopian tube NEC	7E25.00	Therapeutic endoscopic operations on ovary
2695.00	Uterine cervix visualised	4JK2000	High vaginal swab culture positive
7D1Az00	Other repair of vagina NOS	7E04.11	Abdominal hysterectomy
SD13C00	Blister of vulva, infected	2645.00	Vaginal mass
7E18.00	Other repair of fallopian tube	SH74z00	Burn of the vagina or uterus NOS
7D1A600	Abdominal repair vesicovaginal fistula	7E1H400	Operation to ensure patency of fallopian tube NEC
7E00600	Loop diathermy excision of cervix	7N60.00	[SO]Vagina
S77v100	Ovary injury without mention of open wound into cavity	7E1G000	Diagnost endoscop examination & biopsy lesion fallopian tube
7E05.12	Vaginal hysterectomy	K535000	Torsion of the ovary
7D1C000	Evacuation of haematoma from vagina	7D14400	Excision of transverse vaginal septum vertical
1A58000	Vaginal pain	A166z00	Tuberculous oophoritis or salpingitis NOS
7D04100	Reconstruction of vulva with skin graft	7E19.00	Incision of fallopian tube
7D1C.00	Exploration of vagina	7D1B600	Insertion of ring pessary into vagina
7E01500	Colposcopic laser destruction of lesion of cervix	7D04200	Reconstruction of vulva with local flap
7D1By00	Introduction of supporting pessary into vagina OS	K424z00	Other abscess of vulva NOS
7E23y00	Other specified other open operation on ovary	1584.00	Heavy episode of vaginal bleeding
7E11100	Salpingoophorectomy remaining solitary fallop tube and ovary	7E05000	Vaginal hysterocolpectomy and excision of periuterine tissue

4149.00	Cervical cytology sample sent to laboratory
B790.00	Benign neoplasm of cervix uteri NEC
7E22y00	Other specified repair of ovary
7E11300	Salpingectomy of remaining solitary fallopian tube NEC
7D1B300	Change of vaginal pessary
7D1B200	Removal of supporting pessary from vagina
7F22711	Pack to control postnatal vaginal bleeding
SD12D00	Blister of vagina
L246z12	Stenosis of cervix in pregnancy/childbirth/puerperium NOS
685B.00	Ca cervix screen normal
7D1B000	Insertion of Hodge pessary into vagina
B913000	Neoplasm of uncertain behaviour of vagina
L340600	Vaginal tear during delivery
4J17000	High vaginal swab culture
7F06011	McDonald cerclage of cervix
7E14z00	Placement of prosthesis in fallopian tube NOS
7E02500	Diathermy loop cone biopsy of cervix
6856.00	Ca cervix screen - up to date
B440.00	Malignant neoplasm of ovary
L353100	Obstetric laceration of cervix - delivered
4K29000	Cervical smear - borderline change in squamous cells
7B32.00	Vaginal operations to support outlet of female bladder
PC0..00	Anomalies of ovaries
7E20000	Excision of wedge of ovary
K557.11	Polyp of cervix NOS
6854.00	Ca cervix screen - wanted
R150000	[D]Dyskaryotic cervical smear
7D03.00	Extirpation of lesion of vulva
K400000	Acute oophoritis
4K2P.00	Cervical smear - ?non-cervical type glandular neoplasia
685D.00	Ca cervix screen + fee claim
7D1D500	Colposcopic removal of foreign body from vagina
K402z00	Unspecified salpingitis and oophoritis NOS
L247z00	Vaginal abnormality in pregnancy/childbirth/puerperium NOS
7D1A.00	Other repair of vagina
L398500	Delivery by caesarean hysterectomy
K560000	Mild vaginal dysplasia
9O8T.00	Cervical smear slide lost in transit
PC4yC00	Congenital vaginal cyst NEC
PC1y000	Congenital absence of fallopian tube
L340.14	Vaginal tear
685C.00	Ca cervix screen abnormal
SG92.00	Foreign body in vulva and vagina
K551X00	Severe cervical dysplasia, not elsewhere classified
7E0H000	Connection of uterus to vagina
7D1C300	Colposcopy of vagina
A541100	Herpetic vulvovaginitis
4JKE.00	Low vaginal swab taken by patient
7E26000	Diagnostic endoscopic examination and biopsy lesion of ovary
7D15000	Excision of lesion of vagina
K551200	Squamous metaplasia of cervix
4KK3.00	Vaginal vault smear repeat at 24 months
7E1y.00	Other specified operations on fallopian tube
L246412	Stenosis of cervix complicating p/n care - baby deliv prev
7E12400	Left oophorectomy NEC
7E1F200	Endoscopic intrafallopian transfer of gamete
7E01y00	Other specified destruction of lesion of cervix uteri
4K31.00	Cervical smear-no inflammation
7D15800	Excision of vaginal polyp
K552.00	Leukoplakia of cervix
K28yw00	Other tunica vaginalis disease
7D1A100	Repair of urethrovaginal fistula

ZV26211	[V]Fallopian tube insufflation	7D15600	Insertion of vaginal caesium applicators
K402500	Salpingo-oophoritis unspecified	7E16200	Open clipping of remaining solitary fallopian tube
K521400	Sigmoidovaginal fistula	AB21100	Candidiasis of vagina
4K2D.00	Cervical smear transformation zone cells present	7E07000	Dilation cervix uteri & curettage products conception uterus
7D04500	Deinfibulation of vulva	K535.00	Torsion of the ovary, ovarian pedicle or fallopian tube
L39y412	Vaginal discomfort postnatal	L345200	Vulval and perineal haematoma during delivery + p/n problem
AB21z00	Candidal vulvovaginitis NOS	1ABH.11	Vaginal penetration
L247.13	Vaginal abnormality in pregnancy/childbirth/puerperium	K553000	Old laceration of cervix unspecified
7E29100	Transvaginal drainage of ovarian cyst	B58y411	Secondary cancer of the vulva
7E1Hz00	Other operation on fallopian tube NOS	L345.12	Vulval and perineal haematoma during delivery
PC11000	Epoophoron cyst	7E02y00	Other specified biopsy of cervix uteri
7E03z00	Other operation on cervix uteri NOS	K5B5000	Primary vaginal infertility
9O8i.00	Annual cervical smear required	L345.00	Vulval and perineal haematoma during delivery
7D18.00	Other repair of vaginal prolapse	7N60400	[SO]Introitus of vagina
K510.00	Vaginal wall prolapse without uterine prolapse	S886.00	Open wound of vagina
L247300	Vaginal abnormality complicating a/n care-baby not delivered	L245.00	Cervical incompetence
N330600	Postoophorectomy osteoporosis	Z181400	Chaperoning during vaginal examination
PC4y400	Congenital absence of vagina	7E02z00	Biopsy of cervix uteri NOS
SF10200	Crush injury, vulva	L031100	Gravid fallopian tube rupture
7E12000	Salpingoophorectomy NEC	K504000	Endometriosis of the rectovaginal septum
4K26.00	Cervical smear: ? gland neopl.	A980200	Acute gonococcal vulvovaginitis
PC4y.00	Other cervical, vaginal and external female genital anomaly	268A.00	O/E-VE-cervical excit.absent
2698.00	Lesion of cervix	PC41200	Congenital cyst of vulva
7D05z00	Other operation on vulva NOS	12FA.00	FH: Polycystic ovaries
142G.00	H/O: malignant neoplasm ovary	SD15C00	Insect bite, nonvenomous, of vulva, infected
K504.00	Endometriosis of the rectovaginal septum and vagina	7E1Gz11	Laparoscopy of fallopian tube NEC
7H10200	Ligation of patent processus vaginalis	7E04A00	Abdominal hysterectomy with conservation of ovaries
SG92z00	Foreign body in vulva and vagina NOS	A982200	Chronic gonococcal vulvovaginitis
7E23500	Oophorotomy and rupture of cyst	K533200	Acquired atrophy of the fallopian tube
B450z00	Malignant neoplasm of vagina NOS	G856.00	Vulval varices - non obstetric
7E02400	Ring biopsy of cervix uteri	K57y000	Cyst of vulva

685J.00	Vaginal vault smear due	B833700	Vaginal intraepithelial neoplasia grade 1
7D13.00	Other obliteration of vagina	7E25y00	Other specified therapeutic endoscopic operations on ovary
K55y100	Cyst of cervix	S779.00	Injury of ovary
7D19100	Repair of vault of vagina using abdominal approach NEC	PC41.00	Embryonic cyst of cervix/vagina/external female genitalia
7D16700	Vaginoplasty in presence of uterus for absent vagina	7E16511	Open ringing of left fallopian tube
4JK2500	High vaginal swab: white cells seen	1599.00	H/O: hysterectomy
7E01211	Diathermy of lesion of cervix uteri	4K2F.00	Cervical smear pus cells present
7E04100	Abdominal hysterectomy & excision of periuterine tissue NEC	4K3..00	Cervical smear - inflam.change
7F23000	Immed repair obstetric laceration of uterus or cervix uteri	7E05800	Vaginal hysterectomy and left salpingo-oophorectomy
7D18y00	Other specified other repair of vaginal prolapse	K400600	Subacute perioophoritis
K424.00	Other abscess of vulva	K571.00	Atrophy of vulva
7D05100	Drainage of lesion of vulva	7E11700	Left salpingoophorectomy
7E26z00	Diagnostic endoscopic examination of ovary NOS	K533312	Fallopian tube absent acquired
7D03100	Laser destruction of lesion of vulva	7F19000	Manually assisted vaginal delivery
7B32400	Partial removal of tension-free vaginal tape	K570.00	Dystrophy of vulva
7F1B400	Incision of cervix to facilitate delivery	7E14y00	Other specified placement of prosthesis in fallopian tube
K534.00	Prolapse of the ovary and fallopian tube	K56z.00	Noninflammatory vaginal disorder NOS
7D18500	Anterior mesh vaginal repair	7D1B400	Removal of ring pessary from vagina
Pyu6500	[X]Other congenital malformations of vulva	7E00z00	Excision of cervix uteri NOS
8C81.13	Gamete intrafallopian transfer	L354200	Obstetric high vaginal laceration with postnatal problem
7B32100	Introduction of biethium bean through vagina	K5B5z00	Female infertility of vaginal origin NOS
7E20.11	Partial oophorectomy	7E05200	Vaginal hysterocolpectomy NEC
9kF7.11	Endocervical swab culture neg	K521600	Fistula of vagina to small intestine
7E02600	Loop diathermy biopsy of cervix uteri	4K2C.00	Smear NAD - no endocervical cells
685..11	Cervical cytology screen	SE24300	Contusion, vulva
9O8X.00	Cervical smear - suspend recall	7E1B.00	Other open operations on fallopian tube
L34y200	Other vulval/perineal trauma during delivery + p/n problem	R150100	[D] Koilocytosis - cervical smear
7D12y00	Other specified excision of vagina	K534z00	Ovarian and fallopian tube prolapse NOS
L248.13	Vulval abnormality in pregnancy/childbirth/puerperium	4KK..00	Vaginal vault smear action needed
7D14300	Excision of transverse vaginal septum low	K551000	Anaplasia of cervix
K521700	Fistula of vagina to large intestine	S887.00	Open wound of vagina with complication

B7B2z00	Benign neoplasm of vulva NOS	SG92000	Foreign body in vulva
9O83.00	Cervical smear - 3rd call	7B35y11	Spence vaginal urethrocystostomy
Z257.12	Spontaneous vaginal delivery	9O86.00	Cervical smear - 2nd recall
6859.11	Cervical cytology examination	K56y200	Granulation tissue at vaginal vault
7E2By00	Other specified other operations on ovary	7F17200	Vacuum delivery before full dilation of cervix
7D02y00	Other specified excision of vulva	7D0..12	Vulva operations
L247111	Septate vagina - baby delivered	7E23100	Open freeing of adhesions of ovary
7D1D200	Biopsy of lesion of vagina	7D02400	Marsupialisation of lesion of vulva
9O85.00	Cervical smear - 1st recall	Kyu9600	[X]Oth noninflam disorders/ovary,fallopian tbe+broad ligamnt
7E25300	Endoscopic drilling of ovary	7D19000	Repair vaginal vault combined abdominal & vaginal approach
L245000	Cervical incompetence unspecified	7E16300	Open clipping of fallopian tube NEC
7D02.00	Excision of vulva	K562400	Stenosis of vagina
7E01200	Cauterisation of lesion of cervix uteri	A541200	Herpetic ulceration of vulva
Pyu6300	[X]Other congenital malformations of uterus and cervix	7E15100	Open bilateral clipping of fallopian tubes
7E1Cy00	Endoscopic bilateral occlusion of fallopian tubes OS	7D12z00	Excision of vagina NOS
K425100	Ulceration of vulva in diseases EC	SD11C00	Abrasion of vulva, infected
7E16400	Open clipping of right fallopian tube	7D19600	Repair of vault of vagina with mesh using abdominal approach
K533313	Absent ovary, acquired	K562.12	Atresia of vagina
K551100	Epidermidization of cervix	7E01000	Avulsion of lesion of cervix uteri
K520200	Urethrovaginal fistula	PC4yD00	Fusion of vulva
L34z.00	Vulval/perineal trauma during delivery NOS	K561.00	Leukoplakia of vagina
7E2z.00	Ovary and broad ligament operations NOS	7E16600	Open ligation of right fallopian tube
L247.11	Septate vagina in pregnancy, childbirth and the puerperium	7E16y00	Other specified other open occlusion of fallopian tube
PC11z00	Embryonic cyst of fallopian tube or broad ligament NOS	B58y200	Secondary malignant neoplasm of cervix uteri
B41yz00	Malignant neoplasm of other site of cervix NOS	4K2L.00	Cervical smear - high grade dyskaryosis (severe)
7E03011	Lash repair of internal os of cervix	K556.00	Hypertrophic elongation of the cervix
7D17000	Ant and post colporrhaphy and amputation of cervix uteri	4KA..00	Vaginal vault smear result
7E20100	Excision of lesion of ovary	L411.12	Vulval obstetric varicose veins
7D17.00	Repair of vaginal prolapse and amputation of cervix uteri	B833100	Carcinoma in situ of fallopian tube
7E23z00	Other open operation on ovary NOS	K562300	Atresia of vagina
K5B5100	Secondary vaginal infertility	4JRL.00	Cervical cytology screening test

ZV10411	[V]Personal history of malignant neoplasm of cervix uteri	K42y200	Ulcer of vagina
K550.00	Erosion and ectropion of the cervix	L247311	Septate vagina complicating a/n care- baby not yet delivered
268..11	O.E - vaginal exam.- non obst.	PC4y500	Agenesis of vagina
7D1D400	Dilation of vagina	SD11D00	Abrasion of vagina, infected
7E2B000	Transvaginal ultrasound guided aspiration of ovarian cyst	K520700	Vesicovaginal fistula
S884.00	Open wound of vulva	7D04y00	Other specified repair of vulva
7E08900	Vaginal removal of uterine foreign body	7E01700	Loop diathermy of cervix
BB2N.00	[M]Intraepit neop,grade III,of cervix, vulva and vagina	PC11.00	Embryonic cyst of fallopian tube and broad ligament
7E04J00	Subtotl abdominal hysterectomy & right salpingo-oophorectomy	L353z00	Obstetric laceration of cervix NOS
7D1Cz00	Exploration of vagina NOS	L353200	Obstetric laceration of cervix with postnatal problem
4K21.00	Cervical smear:inadequate spec	7E03500	Colposcopic biopsy cervix
7D18z00	Other repair of vaginal prolapse NOS	7D12.00	Excision of vagina
7D1A200	Repair of rectovaginal fistula	4JK9.00	Endocervical chlamydia swab
685I.00	No smear-amputation of cervix	S885.00	Open wound of vulva with complication
62O6.00	Vaginal "show"	7E0Gz00	Other vaginal operation on uterus NOS
B41z.00	Malignant neoplasm of cervix uteri NOS	L353.12	Tear of cervix - obstetric
B7A2.00	Benign teratoma of ovary	B7B0z00	Benign neoplasm of fallopian tube or uterine ligament NOS
B41y.00	Malignant neoplasm of other site of cervix	B41..00	Malignant neoplasm of cervix uteri
8L70.00	Hysterectomy planned	K501.11	Chocolate cyst of ovary
L411.00	VV's of perineum/vulva in pregnancy/puerperium	L353.00	Obstetric laceration of cervix
SD10D00	Abrasion, vagina	K400500	Subacute oophoritis
AB21.11	Monilial vulvovaginitis	4JK2.00	High vaginal swab taken
7E22300	Fixation of ovary NEC	7D1D300	Removal of foreign body from vagina
4K2G.00	Cervical smear red blood cells present	K501.00	Endometriosis of ovary
PC4..00	Cervical, vaginal and external female genital anomalies	S77w100	Ovary injury with open wound into cavity
PC1y.00	Other fallopian tube and broad ligament anomalies	PC40.00	Cervical/vaginal/external female genital anomalies, unspec
K555.00	Incompetence of cervix	K421z00	Vaginitis and vulvovaginitis NOS
7E22000	Replantation of ovary	7D05500	Biopsy of vulva
7E03.00	Other operations on cervix uteri	B833500	Vulval intraepithel neop grd 2
K557.00	Mucous polyp of cervix	7D17.11	Colporrhaphy and amputation of cervix uteri

L246z11	Polyp of cervix in pregnancy/childbirth/puerperium NOS	B7B2.00	Benign neoplasm of vulva
7E04.12	Wertheim hysterectomy	4KK0.00	Vaginal vault smear repeat at 6 months
267..12	O/E - vulva	K516100	Acquired vaginal enterocele
K5B2300	Blocked fallopian tube	K56..00	Noninflammatory vaginal disorders
9O87.00	Cervical smear - 3rd recall	PC43.00	Rectovaginal fistula, congenital
7E0Gy00	Other specified other vaginal operation on uterus	Kyu9B00	[X]Other specified noninflammatory disorders/vulva+perineum
7E1C100	Endoscopic bilateral clipping of fallopian tubes	K534200	Displacement of the ovary and fallopian tube
7E03200	Repair of cervical laceration	L245400	Cervical incompetence with postnatal complication
7M14200	Vaginal approach	7E11900	Left salpingectomy
L031000	Fallopian tube pregnancy	7NB5300	[SO]Vulval vein
7D15300	Cryotherapy to lesion of vagina	7E23300	Open drainage of cyst of ovary
B912.00	Neoplasm of uncertain behaviour of ovary	K425z00	Ulceration of vulva NOS
7E12300	Right oophorectomy NEC	7E1H100	Hydrotubation of fallopian tube
B7A..11	Dermoid cyst of ovary	7D02z00	Excision of vulva NOS
K425.00	Ulceration of vulva	62O6.11	Vaginal "show" - A/N
7D15100	Laser destruction of lesion of vagina	K57..00	Vulval and perineal noninflammatory disorders
15E..00	Vulval irritation	Kyu8400	[X]Ulceration of vulva in infectious+parasitic diseases CE
7E16500	Open clipping of left fallopian tube	7E1F000	Endoscopic freeing of adhesions of fallopian tube
K577100	Moderate vulvar dysplasia	4JK2100	High vaginal swab culture negative
K53z.00	Ovarian, fallopian tube and broad ligament disorder NOS	7D14000	Laser excision of septum of vagina
S885z00	Open wound of vulva with complication, NOS	2691.00	O/E-vaginal speculum exam. NAD
7D15400	Implantation of radioactive substance into vagina	SE24400	Contusion, vagina
5B46.00	Laser cervix lesion therapy	PC4yB00	Atresia of vagina
K424111	Boil of vulva	K5A3000	Atrophy of vagina
K562z11	Vaginal band	S885000	Open wound of vulva with complication, unspecified
9O8g.00	Cervical smear screening telephone invitation	7F1B500	Deinfibulation of vulva to facilitate delivery
7E20.00	Partial excision of ovary	7E1..11	Tubal operations - fallopian
PC4y911	Congenital stricture of vagina	K402400	Perioophoritis unspecified
7D14200	Excision of transverse vaginal septum high	7E11500	Oophorectomy of remaining solitary ovary NEC
279..00	O/E - VE - cervical dilatation	26A..00	O/E - vaginal discharge
1A7..00	Vaginal discharge symptom	7D1C100	Toilet to vagina

L354z00	Obstetric high vaginal laceration NOS	L246211	Polyp of cervix - baby delivered+postpartum complication
B454.11	Primary vulval cancer	K570000	Kraurosis of vulva
7E25z00	Therapeutic endoscopic operation on ovary NOS	Z2A1.00	Vulval toilet
7E04711	Abdominal hysterectomy and left salpingoopherectomy	269A.00	Uterine cervix transformation zone not visualised
7D13z00	Obliteration of vagina NOS	4KA1.00	Vaginal vault smear negative
L354.11	High vaginal laceration - obstetric	L411512	Vaginal varices in pregnancy
K53y400	Infarction of fallopian tube	PC4y411	Rudimentary vagina
7E04600	Radical hysterectomy	K42z.00	Cervical, vaginal and vulval inflammatory disease NOS
159B.00	H/O: bilateral oophorectomy	4JK2400	High vaginal swab: fungal organism isolated
K421.00	Vaginitis and vulvovaginitis	7E00.00	Excision of cervix uteri
K553z00	Old laceration of cervix NOS	7E04F00	Subtotal abdominal hysterectomy with conservation of ovaries
B58y211	Secondary cancer of the cervix	L247211	Septate vagina - baby delivered with postpartum complication
B410.00	Malignant neoplasm of endocervix	Q20y300	Vulval haematoma due to birth trauma
L411z00	Varicose veins of perineum/vulva in pregnancy/puerperium NOS	7E13z11	Partial salpingectomy NEC
9O8..12	Cytology-cervical-admin	PC4y000	Congenital absence of cervix
L247z11	Septate vagina in pregnancy/childbirth/puerperium NOS	K551300	Mild cervical dysplasia
7D12200	Marsupialisation of lesion of vagina	7E14100	Revision of tubal prosthesis in fallopian tube
L248100	Vulval abnormality - baby delivered	7D19y00	Other specified repair of vault of vagina
L345z00	Vulval and perineal haematoma during delivery NOS	B833311	Vulval intraepithelial neoplasia
7E1H300	Insufflation of fallopian tube	SD17C00	Splinter of vulva without major open wound, infected
A913500	Secondary syphilis of vulva	SD1zD00	Superficial injury of vagina NOS, infected
K511z00	Uterine prolapse without vaginal wall prolapse NOS	M181100	Pruritus vulvae
7E04P00	Radical hysterectomy with bilateral salpingo-oophorectomy	7E26y00	Other specified diagnostic endoscopic examination of ovary
9kF9.11	Low vaginal swab culture neg	7E15011	Pomeroy open bilateral ligation of fallopian tubes
7D0z.00	Vulva and female perineum operations NOS	15H..00	Vulval irritation
L247z12	Stenosis of vagina in pregnancy/childbirth/puerperium NOS	PC02.00	Ectopic ovary
7D05y00	Other specified other operation on vulva	268..12	O/E - vaginal examination
7D03500	Painting of vulval warts	K567.00	Polyp of vagina
7D03600	Vulval polypectomy	L247012	Stenosis of vagina affecting obstetric care
7E18200	Anastomosis of fallopian tube NEC	7E1H700	Aspiration of fallopian tube

7E1H200	Dye test of fallopian tube	C164.00	Polycystic ovaries
B440.11	Cancer of ovary	4K4Z.00	Cervical smear action NOS
7E01300	Cryotherapy to lesion of cervix uteri	L340500	Vulval tear during delivery
K502.00	Endometriosis of the fallopian tube	9O8b.00	Cervical smear disclaimer sent
4K4..00	Cervical smear - action needed	Lyu3300	[X]Maternal care for other abnormalities of cervix
PC0z.00	Congenital anomalies of ovaries NOS	K550000	Erosion of cervix
7E21.00	Open destruction of lesion of ovary	15C..00	Vaginal irritation
7E19100	Drainage of fallopian tube	K402.00	Salpingitis and oophoritis unspecified
6859.00	Ca cervix - screen done	4K2A.00	Cervical smear endocervical cells present
K42yz00	Other cervical, vaginal and vulval disease NOS	K513.00	Uterovaginal prolapse, complete
7E08100	Dilation cervix & evacuation products conception uterus NEC	K533300	Acquired absence of ovary or fallopian tube
4K3B.00	Cervical smear - mild inflammation	7F1B300	Manual dilatation of cervix
PC41100	Embryonal cyst of vagina	7E1B300	Exploration of fallopian tube
7E1Gz00	Diagnostic endoscopic examination of fallopian tube NOS	K401.00	Chronic salpingitis and oophoritis
7D1Cy00	Other specified exploration of vagina	65PP.00	Trichomonas vaginalis contact
7E05.00	Vaginal excision of uterus	K42y600	Vulvodynia
4K37.00	Cervical smear - herpes	7E03400	Colposcopy of cervix
4K36.12	HPV changes: cervical smear	K553100	Adhesions of cervix
C164.13	Multicystic ovaries	B831000	Carcinoma in situ of endocervix
7D19500	Sacrospinous fixation of vaginal vault	SG92111	Tampon retained in vagina
7E19y00	Other specified incision of fallopian tube	L34y.00	Other vulval and perineal trauma during delivery
7E1Gy00	Diagnostic endoscopic examination of fallopian tube OS	K562000	Post-operative vaginal adhesions
K533.00	Acquired atrophy of the ovary and fallopian tube	Z263D00	Intact membranes bulging through cervix
7B32300	Total removal of tension-free vaginal tape	7E05500	Vaginal hysterectomy with conservation of ovaries
7E18100	Replantation of fallopian tube	L247000	Vaginal abnormality affecting obstetric care
981..11	Cervical smear - fee claim	ZG52100	Advice on cervical cytology
41F0.00	Endocervical swab	PC1yz00	Other fallopian tube or broad ligament anomalies NOS
7D11.00	Other operations on introitus of vagina	R150.00	[D]Nonspecific abnormal Papanicolaou cervical smear
7D10z00	Incision of introitus of vagina NOS	278..00	O/E - VE - cervix ripeness
4K33.00	Cervical smear - trichomonas	K5E..00	Other abnormal uterine and vaginal bleeding
7E12100	Salpingectomy NEC	1AE..00	Vaginal discomfort

B833000	Carcinoma in situ of ovary	7E17111	Open removal of ring from fallopian tube NEC
K574000	Old laceration of vulva	Pyu6400	[X]Other congenital malformations of vagina
4K4E.00	Cervical smear repeat at 24 months	K554z00	Stricture and stenosis of cervix NOS
7E1Fy00	Other therapeutic endoscopic operation on fallopian tube OS	L248.00	Congenital/acquired abnormality vulva in preg/childb/puerp
4K2R.00	Cervical smear - human papillomavirus positive	PC1y100	Accessory fallopian tube
K516.00	Vaginal enterocele	R128.00	[D]Vaginal fluid abnormal
7E1F100	Endoscopic injection into fallopian tube	7E23000	Transposition of ovary
4K34.00	Cervical smear - candida	Kyu9K00	[X]Severe cervical dysplasia, not elsewhere classified
2696.00	360 degree sweep of cervix performed	7E1D.00	Other endoscopic occlusion of fallopian tube
7D14.00	Excision of band of vagina	4KA3.00	Vaginal vault smear-atrophic
7E07011	Dilation cervix uteri & curettage for termination pregnancy	L34..12	Vulval delivery trauma
4K2K.00	Cervical smear - high grade dyskaryosis (moderate)	L246.12	Stenosis of cervix in pregnancy, childbirth, puerperium
142E.00	H/O: malignant neoplasm cervix	4JK7.00	Vaginal swab culture positive
L296.00	Vaginal delivery following previous caesarean section	K554200	Occlusion of cervix
7D14100	Excision of septum of vagina NEC	K554.00	Stricture and stenosis of cervix
Ayu4L00	[X]Vulval warts	B450100	Malignant neoplasm of vaginal vault
K560100	Moderate vaginal dysplasia	1V05.00	Misuses drugs vaginally
7E23400	Oophorotomy and drainage of abscess	15J..00	Vulval rash
4K2Z.00	Cervical smear result NOS	K562200	Occlusion of vagina
4K2E.00	Cervical smear transformation zone cells absent	K42..00	Cervical, vaginal and vulval inflammatory diseases
L20..11	Spontaneous vaginal delivery	K560.00	Dysplasia of vagina
7D0y.00	Other specified operations on vulva or female perineum	PC1..00	Fallopian tube and broad ligament anomalies
4K2B.00	Cervical smear endocervical cells absent	7D19200	Repair of vault of vagina using vaginal approach NEC
4K3Z.00	Cervical smear - inflam. NOS	PC00.11	Agenesis of ovary
9O8V.00	Place cervical smear taken	Kyu8600	[X]Vulvovaginal ulceration+inflammation in other diseases CE
7E00100	Wedge excision of cervix uteri and suture HFQ	685Z.00	Ca cervix screen NOS
7E01411	Polypectomy of cervix	7F23200	Immed repair obstetric laceration vagina and floor of pelvis
B48y100	Malignant neoplasm of tunica vaginalis	AD10100	Trichomonal vulvovaginitis
L247212	Stenosis of vagina - baby delivered+postpartum complication	7E26.00	Diagnostic endoscopic examination of ovary
7E1F.11	Other therapeutic laparoscic operations on fallopian tube	9O8d.00	Cervical smear screening second letter
SP07900	Problem with vaginal pessary	K551.00	Dysplasia of cervix uteri

7E1E000	Endoscopic removal of clip from fallopian tube	7E12200	Oophorectomy NEC
7D17100	Anterior colporrhaphy and amputation of cervix uteri NEC	K400.00	Acute salpingitis and oophoritis
ZV26112	[V]Gamete intrafallopian transfer	7E26.11	Laparoscopy of ovary
PC10.00	Fallopian tube and broad ligament anomalies, unspecified	PC01.00	Accessory ovary
7E04E00	Laparoscopic subtotal hysterectomy	9kF9.00	Low vaginal swab culture negative - enhanced services admini
K535z00	Ovary, ovarian pedicle or fallopian tube torsion NOS	L245100	Cervical incompetence - delivered
Kyu9900	[X]Other specified noninflammatory disorders/cervix uteri	PC4y600	Congenital absence of vulva
7E1Dz00	Endoscopic occlusion of fallopian tube NOS	7D17200	Posterior colporrhaphy and amputation of cervix uteri NEC
7E22z00	Repair of ovary NOS	B410000	Malignant neoplasm of endocervical canal
B441.00	Malignant neoplasm of fallopian tube	9Ndx.00	Informed consent for cervical smear given
7E05.11	Schauta radical vaginal hysterectomy	4K38.00	Cervical smear - actinomyces
7E18z00	Repair of fallopian tube NOS	9O8a.00	Cervical smear every 12 months for life
7D1B500	Renewal of supporting pessary in vagina	K57z.00	Noninflammatory vulval and perineal disorder NOS
7D10.00	Incision of introitus of vagina	K56yz00	Other noninflammatory vaginal disorder NOS
4K4D.00	Cervical smear repeat at 48 months	B831.11	CIN III - carcinoma in situ of cervix
4JK6.00	Vaginal swab culture negative	L247312	Stenosis of vagina complicating a/n care- baby not delivered
4K2..11	Dyskaryosis on cervical smear	S884z00	Open wound of vulva, NOS
L246311	Polyp of cervix complicating a/n care- baby not delivered	L39y411	Postnatal vaginal discomfort
K553.00	Old laceration of cervix	7D17z00	Repair of vaginal prolapse & amputation of cervix uteri NOS
4K32.00	Cervical smear-severe inflamm.	K421200	Vulvovaginitis unspecified
7E05300	Vaginal hysterectomy NEC	7D04411	Reconstruction of vulva with free flap
4J17100	Low vaginal swab culture	B58y300	Secondary malignant neoplasm of vagina
7E26011	Laparoscopic biopsy of ovary	ZV13D00	[V]Personal history of severe cervical dysplasia
K424200	Furuncle of vulva	K574100	Old scarring of vulva
7E20y00	Other specified partial excision of ovary	9O8W.00	Cervical smear to continue post hysterectomy
B58y400	Secondary malignant neoplasm of vulva	7C22000	Construction of penis
B410100	Malignant neoplasm of endocervical gland	K56y.00	Other noninflammatory vaginal disorders
7D19011	Zacharin repair of vaginal vault	4K22.00	Cervical smear: negative
14NF.00	H/O: defibulation of vulva	S884000	Open wound of vulva, unspecified
L248000	Vulval abnormality affecting obstetric care	7E1H.00	Other operations on fallopian tube
7D1D000	Freeing of adhesions of vagina	N331200	Postoophorectomy osteoporosis with pathological fracture

McKechnie DGJ, *et al. BMJMED* 2023; 2:e000499. doi: 10.1136/bmjmed-2023-000499

7E1C300	Endoscopic bilateral placement of intrafallopian implants	K401000	Chronic oophoritis
7E1C200	Endoscopic bilateral ringing of fallopian tubes	K56y111	Bleeding - vaginal NOS
A166111	Fallopian tube tuberculosis	Z257.13	SVD - Spontaneous vaginal delivery
7D05000	Biopsy of lesion of vulva	SD12C00	Blister of vulva
PC4yA00	Atresia of cervix	K577.00	Dysplasia of vulva
7E2..00	Ovary and broad ligament operations	SP0D100	Hyperstimulation of ovaries
7E05z00	Vaginal excision of uterus NOS	K512.00	Uterovaginal prolapse, incomplete
7B33A00	Creation of urethrovaginal fistula	SD14C00	Insect bite, nonvenomous, of vulva
7E18y00	Other specified repair of fallopian tube	L34..00	Trauma to perineum and vulva during delivery
L411513	Vulval varices in pregnancy	268..00	O/E - bi-manual vaginal exam.
7E02000	Knife cone biopsy of cervix uteri	7E05600	Lap assist vag hysterectomy with bilat salpingo-oophorectomy
7D17111	Fothergill anterior colporrhaphy and amputation of cervix	7D1B100	Insertion of ring into vagina
7E13000	Excision of lesion of fallopian tube	K400100	Acute perioophoritis
7E1C000	Endoscopic bilateral cauterisation of fallopian tubes	ZV76200	[V]Screening for malignant neoplasm of cervix
L34zz00	Vulval/perineal trauma during delivery NOS	4K36.11	Herpes: cervical smear
7E05700	Vaginal hysterectomy and right salpingo-oophorectomy	7D03200	Cryosurgery of lesion of vulva
K550211	Ectopy of cervix	K553200	Cicatrix (postpartum) of cervix
L34z100	Vulval/perineal trauma during delivery NOS - delivered	7E25100	Endoscopic freeing of adhesions of ovary
PC03.00	Streak ovary	K551z00	Dysplasia of cervix NOS
7E04500	Abdominal hysterectomy and bilateral salpingoophorectomy	L34yz00	Other vulval/perineal trauma during delivery NOS
8CEA.00	Cervical smear information leaflet given	6851.00	Ca cervix screen - not offered
7E1Fz00	Other therapeutic endoscopic operation on fallopian tube NOS	7F18.00	Cephalic vaginal deliv abnorm presentation head - no instrum
6793.00	Health ed. - cervical cytology	B7B0.00	Benign neoplasm of fallopian tubes and uterine ligaments
7E1G.00	Diagnostic endoscopic examination of fallopian tube	7E19z00	Incision of fallopian tube NOS
7E21z00	Open destruction of lesion of ovary NOS	4KA2.00	Vaginal vault smear-inadequate
7E1Bz00	Open operation on fallopian tube NOS	7E1F.00	Other therapeutic endoscopic operations on fallopian tube
K566.00	Vaginal haematoma	7N60211	[SO]Skin of vulva
4K4B.00	Cervical smear repeat at 36 months	Z246500	Time vaginal show detected
S77v000	Fallopian tube injury without open wound into cavity	K425000	Ulceration of vulva unspecified
SD17D00	Splinter of vagina without major open wound, infected	Q41y111	Perinatal transient vaginal bleeding
7D19.00	Repair of vault of vagina	7E1G.11	Laparoscopy of fallopian tube

7E1B200	Open dilation of fallopian tube
7E05100	Vaginal hysterectomy and excision of periuterine tissue NEC
4JKD.00	Low vaginal swab for chlamydia taken by patient
7E11600	Right salpingoophorectomy
7D1D.00	Other operations on vagina
K515.00	Post hysterectomy vaginal vault prolapse
K576.00	Polyp of labia and vulva
K520100	Ureterovaginal fistula
K562.00	Stricture or atresia of the vagina
7B30z00	Combined abdo & vaginal op to support outlet fem bladder NOS
AD10011	Flour vaginalis - trichomonal
K55y400	Hypertrophy of cervix
K577W00	Dysplasia of vulva, unspecified
7E14200	Removal of tubal prosthesis from fallopian tube
K570z00	Dystrophy of vulva NOS
7D10y00	Other specified incision of introitus of vagina
7E04400	Subtotal abdominal hysterectomy
7E16100	Open ligation of fallopian tube NEC
B454.00	Malignant neoplasm of vulva unspecified
7D04400	Reconstruction of vulva with microvascular transfered flap
ZV76212	[V]Routine cervical smear
L245300	Cervical incompetence with antenatal problem
14NF000	H/O: defibulation of vulva to facilitate delivery
B411.00	Malignant neoplasm of exocervix
7E04512	TAH - total abdom hysterectomy & bilateral salpingoophorect
K42y.00	Other cervical, vaginal and vulval diseases
7D1D211	Biopsy of vaginal wall
7E0G000	Vaginal excision of lesion of uterus
7D04000	Unspecified repair of vulva
7D04.11	Reconstruction of vulva

7E11800	Right salpingectomy
K5B5.00	Female infertility of vaginal origin
L247100	Vaginal abnormality - baby delivered
K53y500	Rupture of fallopian tube
7D03211	Cryotherapy of lesion of vulva
7E1..00	Fallopian tube operations
7D05.00	Other operations on vulva
K5Ez.00	Abnormal uterine and vaginal bleeding, unspecified
7B32z00	Vaginal operation to support outlet of female bladder NOS
L354.12	High vaginal tear - obstetric
K425200	Ulceration of vulva in Behcet's disease
L411400	VV's of perineum/vulva in pregnancy/puerperium + p/n comp
7E02100	Laser cone biopsy of cervix uteri
L245z00	Cervical incompetence NOS
Kyu8500	[X]Vaginits,vulvits+vulvovaginitis/infect+parasitc diseas CE
ZV25600	[V]Failed interruption of fallopian tubes
7E0G.00	Other vaginal operations on uterus
7E23200	Open biopsy of lesion of ovary
L245200	Cervical incompetence - delivered with postnatal comp
7D1A000	Unspecified repair of vesicovaginal fistula
1A71.00	Vaginal discharge absent
7E1By00	Other specified open operation on fallopian tube
7D19400	Suspension of vagina NEC
K42y000	Carbuncle of vagina
B833400	Vulval intraepithel neop grd 1
7E1Hy00	Other specified other operation on fallopian tube
K400200	Acute salpingo-oophoritis
L248200	Vulval abnormality - baby delivered+postpartum complication
PC05.00	Congenital torsion of ovary
7E1C.00	Endoscopic bilateral occlusion of fallopian tubes

7D04.00	Repair of vulva
PC00.00	Congenital absence of ovary
ZX16400	Gouging own vagina
7E1D200	Endoscopic occlusion of left fallopian tube
K535200	Torsion of the fallopian tube
B7B1z00	Benign neoplasm vagina NOS
7D03z00	Extirpation of lesion of vulva NOS
7D03y00	Other specified extirpation of lesion of vulva
K55y000	Senile atrophy of cervix
7E16211	Open clipping of residual solitary fallopian tube
7D15200	Cauterisation of lesion of vagina
7B32000	Vaginal buttressing of urethra
B7B1000	Skin tag in vagina
K550100	Ulcer of cervix
K521200	Rectovaginal fistula
K576z00	Polyp of labia and vulva NOS
7E04H00	Subtotl abdominal hysterectomy & bilat salpingo-oophorectomy
L03y000	Cervical pregnancy
K554.11	Stenosis of cervix uteri
K56X.00	Severe vaginal dysplasia, not elsewhere classified
K530.00	Follicular cyst of ovary
7E02011	Sturmdorf conisation of cervix uteri
7D03300	Cauterisation of lesion of vulva
Q414300	Neonatal vaginal haemorrhage
K562.11	Adhesions of vagina
1A58100	Vulval pain
7E06y11	Vaginal myomectomy
7E1H411	Dilation of fallopian tube NEC
7D03011	Biopsy of vulva
6858.00	Ca cervix screen - not reached
K50y300	Endometriosis of the vulva
K41..00	Uterine inflammatory diseases excluding the cervix
Q476.11	Patent processus vaginalis
7E02300	Punch biopsy of cervix uteri
1591.00	H/O: recurrent vaginal disch.
7D05600	Vulvoscopy
SD1yC00	Superficial injury of vulva NOS, without major open wound
B831.00	Carcinoma in situ of cervix uteri
7E2A211	Cervical smear NEC
7N60200	[SO]Vulva
K574.00	Old laceration or scarring of vulva
7E04K00	Subtotal abdominal hysterectomy & left salpingo-oophorectomy
K400.11	Oophoritis - acute
7E2Bz00	Other operations on ovary NOS
L34z200	Vulval/perineal trauma during delivery NOS with p/n problem
K504z00	Endometriosis of the rectovaginal septum and vagina NOS
4K2Q.00	Cervical smear - human papillomavirus negative
7E07200	Dilation of cervix uteri and curettage of uterus NEC
7D1Ay00	Other specified other repair of vagina
7N62100	[SO]Fallopian tube
7D13y00	Other specified obliteration of vagina
PC1z.00	Fallopian tube or broad ligament anomalies NOS
PC41z00	Embryonic cyst cervix/vagina/external female genitalia NOS
L092300	Cervix damage following abortive pregnancy
L345000	Vulval and perineal haematoma during delivery, unspecified
7E14000	Insertion of tubal prosthesis into fallopian tube
K55y200	Fibrosis of cervix
B833200	Carcinoma in situ of vagina
L353.11	Laceration of cervix - obstetric
K532000	Corpus albicans cyst of the ovary

7E01600	Cold coagulation of lesion of cervix	7E1H600	Recanalisation of fallopian tube
7D03000	Excision of lesion of vulva NEC	7D1Bz00	Introduction of supporting pessary into vagina NOS
981..00	FP74 - cervical cytology claim	L247.12	Stenosis of vagina in pregnancy/childbirth/puerperium
7E04900	TAH - Tot abdom hysterectomy and BSO - bilat salpingophorect	7D15z00	Extirpation of lesion of vagina NOS
SD13D00	Blister of vagina, infected	L354100	Obstetric high vaginal laceration - delivered
L246411	Polyp of cervix complicating p/n care - baby deliv prev	K423.11	Vulvovaginal gland abscess
4K2H.00	Cervical smear epithelial cells absent	9EVC.00	Cervical smear report received
7E04B00	Lapar total abdominal hysterect bilat salpingo-oophorectomy	PC41300	Embryonic cyst of cervix
B586.00	Secondary malignant neoplasm of ovary	685H.11	No smear - hysterectomy
PC0y.00	Other specified congenital anomalies of ovaries	8572.00	Vibration of cervix
K533z00	Acquired atrophy of the ovary and fallopian tube NOS	K400700	Subacute salpingo-oophoritis
7E04C00	Laparoscopic hysterectomy	7D1A500	Vaginal repair of vesicovaginal fistula
K421111	Vulval sores	S77A.00	Injury of fallopian tube
K504100	Endometriosis of the vagina	1ABH.00	Vaginal penetrative sexual intercourse
K550200	Ectropion (eversion) of cervix	K57y200	Stricture of vulva
L354000	Obstetric high vaginal laceration unspecified	7E23700	Open diagnostic aspiration of ovary
7E16411	Open ringing of right fallopian tube	PC4y100	Agenesis of cervix
K500200	Endometriosis of cervix	Z254900	Vaginal delivery
PC4yw00	Other congenital anomaly of vagina	58D3.00	Transvaginal ultrasound scan
K424100	Carbuncle of vulva	7E17100	Open removal of clip from fallopian tube NEC
7E23600	Oophorotomy	BB80200	[M]Borderline mucinous cystadenoma of the ovary
14I..00	H/O abnormal cervical smear	L353000	Obstetric laceration of cervix unspecified
7B32200	Introduction of tension free vaginal tape	1A7Z.00	Vaginal discharge NOS
B833800	Vaginal intraepithelial neoplasia grade 2	7E24300	Transvaginal oocyte recovery
7E04311	Bonney abdominal hysterectomy	K576100	Polyp of vulva
K574z00	Old laceration or scar of vulva NOS	7E16.12	Unilateral occlusion of fallopian tube
Kyu9A00	[X]Other specified noninflammatory disorders of vagina	278Z.00	O/E - VE - cervix ripeness NOS
1AD..00	Vaginal dryness	7E02200	Cone biopsy of cervix uteri NEC
7E16212	Open ringing of remaining solitary fallopian tube	K401z00	Chronic salpingitis and oophoritis NOS
7E04300	Total abdominal hysterectomy NEC	7D03312	Diathermy of vulval lesion

L248400	Vulval abn complicating p/n care - baby delivered previously
4JK..11	Vaginal swab taken
B450.00	Malignant neoplasm of vagina
7E00200	Excision of lesion of cervix uteri
B7A..00	Benign neoplasm of ovary
7E25200	Endoscopic drainage of cyst of ovary
K5E2.00	Abnormal vaginal bleeding, unspecified
K521100	Intestinovaginal fistula
7D04z00	Repair of vulva NOS
K533100	Senile involution of the ovary
4K3C.00	Cervical smear - moderate inflammation
K577X00	Severe vulvar dysplasia, not elsewhere classified
K57y.00	Other noninflammatory vulval and perineal disorders
A166000	Tuberculous oophoritis
K532300	Simple cystoma of the ovary
7E15.00	Open bilateral occlusion of fallopian tubes
L247400	Vaginal abnormality complicating p/n care - baby deliv prev
7D1Dy00	Other specified other operation on vagina
K550z00	Erosion and ectropion of the cervix NOS
L411100	VV's of perineum/vulva in pregnancy/puerperium - delivered
7E14.00	Placement of prosthesis in fallopian tube
7F06100	Removal of cerclage from cervix of gravid uterus
L411000	VV's of perineum/vulva in pregnancy/puerperium unspecified
AB21z11	Candidiasis cervix
2782.00	O/E - VE - cervix ripe
Pyu6000	[X]Other congenital malformations of ovary
7E1D000	Endoscopic occlusion of remaining solitary fallopian tube
67DA.00	Provision of information about cervical screening programme
L092600	Vaginal damage following abortive pregnancy
7E13y00	Other specified partial excision of fallopian tube
7E03y00	Other specified other operation on cervix uteri
7B30.00	Combin abdominal & vaginal ops support outlet female bladder
L34z000	Vulval/perineal trauma during delivery NOS unspec
K565.12	Vaginal discharge NOS
4KA4.00	Vaginal vault smear abnormal
4K55.00	Cervical cytology test
7E01z00	Destruction of lesion of cervix uteri NOS
7E16000	Open ligation of remaining solitary fallopian tube
7E15111	Open bilateral ringing of fallopian tubes
K521300	Rectovulval fistula
7E21000	Open cauterisation of lesion of ovary
SH74.00	Burn of the vagina and uterus
L354.00	Obstetric high vaginal laceration

Year	Rate of newly-recorded transgender identity coding, per 100,000 person years (95% confidence interval)							
	Age group							
	10-12	13-15	16-17	18-29	30-39	40-49	50+	All age groups
2000	2.84 (0.34-10.27)	-	-	3.02 (1.38-5.73)	2.53 (1.16-4.81)	1.92 (0.70-4.17)	0.25 (0.03-0.92)	1.45 (0.96-2.10)
2001	-	-	4.01 (0.49-14.47)	1.43 (0.46-3.33)	1.41 (0.52-3.06)	1.84 (0.74-3.79)	0.84 (0.36-1.66)	1.21 (0.80-1.74)
2002	-	-	1.64 (0.04-9.15)	0.48 (0.06-1.72)	0.80 (0.22-2.04)	1.53 (0.62-3.15)	0.27 (0.06-0.78)	0.62 (0.36-0.99)
2003	-	0.91 (0.02-5.05)	-	1.49 (0.60-3.07)	2.36 (1.26-4.04)	1.16 (0.43-2.53)	0.24 (0.05-0.70)	0.97 (0.66-1.39)
2004	0.79 (0.02-4.42)	0.81 (0.02-4.50)	1.27 (0.03-7.05)	2.12 (1.06-3.8)	1.52 (0.70-2.89)	1.22 (0.49-2.51)	0.29 (0.08-0.75)	1.00 (0.70-1.40)
2005	-	-	1.18 (0.03-6.59)	1.64 (0.75-3.11)	1.32 (0.57-2.61)	0.81 (0.26-1.90)	0.62 (0.28-1.18)	0.90 (0.61-1.27)
2006	-	1.48 (0.18-5.34)	2.27 (0.27-8.20)	1.05 (0.39-2.29)	0.83 (0.27-1.94)	1.71 (0.86-3.07)	0.47 (0.19-0.96)	0.90 (0.62-1.26)
2007	0.71 (0.02-3.95)	0.72 (0.02-4.03)	-	2.35 (1.28-3.94)	0.67 (0.18-1.70)	0.90 (0.33-1.96)	0.52 (0.22-1.02)	0.90 (0.62-1.26)
2008	-	-	2.12 (0.26-7.67)	0.81 (0.26-1.89)	0.67 (0.18-1.72)	0.73 (0.24-1.71)	0.63 (0.30-1.16)	0.67 (0.44-0.99)
2009	-	1.40 (0.17-5.05)	6.36 (2.33-13.85)	2.22 (1.22-3.73)	1.69 (0.81-3.11)	1.16 (0.50-2.29)	0.37 (0.14-0.82)	1.18 (0.86-1.57)
2010	-	0.71 (0.02-3.96)	3.27 (0.68-9.57)	3.25 (1.99-5.02)	2.46 (1.34-4.12)	1.78 (0.92-3.12)	0.57 (0.26-1.08)	1.55 (1.18-2.00)
2011	0.69 (0.02-3.85)	1.39 (0.17-5.03)	10.75 (5.16-19.77)	3.21 (1.96-4.96)	1.41 (0.61-2.77)	2.66 (1.58-4.21)	0.44 (0.17-0.90)	1.71 (1.32-2.18)
2012	0.70 (0.02-3.88)	1.37 (0.17-4.94)	10.65 (5.10-19.58)	4.29 (2.83-6.24)	3.01 (1.75-4.82)	1.78 (0.92-3.12)	0.55 (0.25-1.05)	2.01 (1.59-2.51)
2013	0.72 (0.02-4.04)	6.39 (2.92-12.13)	14.22 (7.57-24.32)	3.79 (2.40-5.69)	1.85 (0.89-3.40)	1.72 (0.86-3.08)	0.25 (0.07-0.65)	1.90 (1.48-2.40)
2014	0.77 (0.02-4.27)	7.58 (3.63-13.94)	17.2 (9.63-28.38)	7.52 (5.44-10.13)	3.75 (2.25-5.85)	1.70 (0.81-3.12)	0.46 (0.19-0.96)	2.98 (2.43-3.60)
2015	4.38 (1.42-10.23)	22.45 (14.53-33.14)	37.39 (24.84-54.04)	15.82 (12.49-19.78)	2.76 (1.43-4.83)	2.24 (1.12-4.01)	1.54 (0.94-2.38)	5.92 (5.08-6.85)
2016	7.34 (2.95-15.13)	30.61 (20.34-44.24)	48.87 (32.97-69.77)	17.31 (13.47-21.91)	3.33 (1.72-5.81)	4.08 (2.33-6.62)	0.84 (0.39-1.60)	6.93 (5.93-8.05)
2017	2.47 (0.30-8.92)	33.70 (22.01-49.37)	48.59 (31.45-71.73)	15.23 (11.27-20.14)	2.99 (1.37-5.68)	3.72 (1.92-6.50)	0.77 (0.31-1.59)	6.31 (5.27-7.49)
2018	7.01 (2.28-16.36)	37.67 (24.61-55.20)	78.39 (54.60-109.02)	18.69 (13.88-24.65)	3.92 (1.88-7.22)	2.92 (1.26-5.75)	0.74 (0.27-1.61)	7.81 (6.57-9.22)

Supplementary table 4: rates of first-recorded transgender identity, by age group and year, between 2000-2018. "-" indicates a rate of zero.

Year	Proportion with recorded transgender identity, per 10,000 (95% confidence interval)							
	Age group							
	10-12	13-15	16-17	18-29	30-39	40-49	50+	All age groups
2000	-	0.20 (0.01-1.11)	-	0.73 (0.41-1.21)	1.15 (0.78-1.65)	1.25 (0.84-1.78)	0.34 (0.21-0.53)	0.68 (0.55-0.83)
2001	-	0.30 (0.04-1.07)	-	0.96 (0.63-1.41)	1.16 (0.83-1.59)	1.41 (1.03-1.89)	0.47 (0.33-0.65)	0.80 (0.68-0.94)
2002	0.17 (0-0.95)	0.24 (0.03-0.87)	0.19 (0-1.07)	0.8 (0.52-1.17)	1.23 (0.92-1.62)	1.49 (1.14-1.93)	0.53 (0.40-0.70)	0.83 (0.72-0.96)
2003	-	0.10 (0-0.57)	0.49 (0.10-1.42)	0.87 (0.60-1.22)	1.52 (1.19-1.92)	1.67 (1.31-2.08)	0.64 (0.50-0.80)	0.96 (0.85-1.09)
2004	-	-	0.71 (0.23-1.65)	0.79 (0.55-1.1)	1.64 (1.31-2.03)	1.73 (1.39-2.12)	0.73 (0.59-0.89)	1.02 (0.91-1.14)
2005	0.12 (0-0.67)	-	0.25 (0.03-0.91)	0.83 (0.60-1.14)	1.70 (1.37-2.09)	1.89 (1.55-2.27)	0.86 (0.71-1.03)	1.11 (1.00-1.23)
2006	0.12 (0-0.65)	0.08 (0-0.43)	0.24 (0.03-0.86)	1.14 (0.87-1.48)	1.76 (1.43-2.15)	2.02 (1.68-2.41)	0.97 (0.82-1.15)	1.24 (1.12-1.36)
2007	0.11 (0-0.63)	0.15 (0.02-0.55)	0.23 (0.03-0.83)	1.12 (0.85-1.44)	2.03 (1.67-2.45)	2.04 (1.70-2.42)	1.08 (0.92-1.26)	1.33 (1.21-1.45)
2008	0.11 (0-0.60)	0.22 (0.05-0.65)	0.22 (0.03-0.81)	1.08 (0.83-1.40)	1.87 (1.53-2.27)	2.16 (1.82-2.55)	1.23 (1.06-1.42)	1.38 (1.26-1.51)
2009	0.21 (0.03-0.77)	0.37 (0.12-0.86)	0.68 (0.25-1.49)	1.29 (1.01-1.63)	1.74 (1.41-2.14)	2.43 (2.07-2.84)	1.32 (1.15-1.52)	1.50 (1.38-1.63)
2010	0.21 (0.03-0.78)	0.22 (0.05-0.65)	0.69 (0.25-1.49)	1.40 (1.11-1.75)	2.14 (1.77-2.58)	2.70 (2.31-3.13)	1.48 (1.30-1.69)	1.69 (1.55-1.83)
2011	0.33 (0.07-0.97)	0.29 (0.08-0.75)	1.73 (0.97-2.85)	1.81 (1.47-2.21)	2.33 (1.92-2.78)	2.98 (2.56-3.43)	1.61 (1.41-1.82)	1.91 (1.76-2.06)
2012	0.33 (0.07-0.97)	0.43 (0.16-0.94)	1.91 (1.11-3.06)	2.42 (2.03-2.86)	2.45 (2.04-2.91)	2.76 (2.36-3.20)	1.71 (1.51-1.93)	2.03 (1.88-2.18)
2013	0.59 (0.19-1.37)	0.69 (0.32-1.32)	2.13 (1.26-3.36)	3.09 (2.64-3.61)	2.28 (1.88-2.75)	2.95 (2.52-3.42)	1.76 (1.56-1.99)	2.19 (2.03-2.35)
2014	0.51 (0.14-1.30)	1.27 (0.71-2.10)	1.78 (0.97-2.99)	3.91 (3.37-4.51)	2.68 (2.22-3.22)	2.94 (2.49-3.44)	1.87 (1.65-2.11)	2.42 (2.25-2.60)
2015	0.62 (0.17-1.58)	1.90 (1.12-3.00)	6.54 (4.72-8.85)	5.98 (5.24-6.80)	2.45 (1.96-3.01)	3.29 (2.76-3.90)	1.86 (1.61-2.13)	2.91 (2.70-3.13)
2016	0.35 (0.04-1.27)	4.35 (3.04-6.02)	8.95 (6.64-11.79)	8.28 (7.35-9.31)	2.82 (2.27-3.46)	3.17 (2.61-3.82)	2.08 (1.80-2.39)	3.55 (3.30-3.81)
2017	0.79 (0.22-2.03)	5.94 (4.28-8.02)	11.06 (8.26-14.51)	9.65 (8.54-10.87)	3.80 (3.11-4.61)	3.50 (2.86-4.25)	2.09 (1.79-2.43)	4.05 (3.77-4.35)
2018	1.07 (0.35-2.49)	5.86 (4.15-8.04)	16.23 (12.60-20.57)	12.42 (11.06-13.90)	4.62 (3.8-5.58)	3.25 (2.59-4.03)	2.3 (1.98-2.66)	4.71 (4.38-5.05)

Supplementary table 5: proportion of people with recorded transgender identity, by age group and year, between 2000-2018. "-" indicates a proportion of zero.

	**Rate of newly-recorded codes, per 100,000 person-years (95% CI)**	**Incidence rate ratio* (95% CI)**	**Proportion of people with transgender identity, per 10,000 (95% CI)**	**Prevalence rate ratio* (95% CI)**
**Age group (years)** ***[between 2000-2009 only]***				
10-12	0.33 (0.09-0.85)	0.17 (0.06-0.51)	0.10 (0.04-0.2)	0.29 (0.12-0.68)
13-15	0.60 (0.24-1.24)	0.30 (0.12-0.74)	0.16 (0.09-0.26)	0.48 (0.26-0.90)
16-17	1.99 (1.11-3.28)	1	0.34 (0.21-0.5)	1
18-29	1.63 (1.3-2.03)	0.78 (0.45-1.36)	1.00 (0.91-1.1)	2.73 (1.80-4.16)
30-39	1.33 (1.04-1.67)	0.67 (0.38-1.17)	1.64 (1.52-1.76)	4.87 (3.22-7.38)
40-49	1.23 (0.95-1.56)	0.65 (0.37-1.14)	1.90 (1.78-2.02)	5.87 (3.88-8.87)
50+	0.46 (0.35-0.59)	0.24 (0.14-0.43)	0.89 (0.84-0.95)	2.83 (1.88-4.29)

**adjusted for age group, Townsend deprivation score, and calendar year*
95% CI = 95% confidence interval

Supplementary table 6: Rates of new recording and proportions of transgender identity per age group, between 2000-2009.

McKechnie DGJ, *et al. BMJMED* 2023; 2:e000499. doi: 10.1136/bmjmed-2023-000499
